# Convergent evolution of a parasite-encoded complement control protein-scaffold to mimic binding of mammalian TGF-β to its receptors, TβRI and TβRII

**DOI:** 10.1016/j.jbc.2022.101994

**Published:** 2022-04-29

**Authors:** Ananya Mukundan, Chang-Hyeock Byeon, Cynthia S. Hinck, Kyle Cunningham, Tiffany Campion, Danielle J. Smyth, Rick M. Maizels, Andrew P. Hinck

**Affiliations:** 1 Department of Structural Biology, University of Pittsburgh School of Medicine, Pittsburgh, Pennsylvania USA; 2 Wellcome Centre for Integrative Parasitology, Institute of Infection, Immunity and Inflammation, University of Glasgow, Glasgow, United Kingdom

**Keywords:** TGF-β, TGF-β mimic, *H.polygyrus*, parasite, regulatory T cell, immune suppression, nuclear magnetic resonance, isothermal titration calorimetry, surface plasmon resonance, TGM, CCP, complement control protein, CSP, chemical shift perturbation, HES, *H. polygyrus* excretory-secretory product, *Hp*ARI, *H. polygyrus* Alarmin Release Inhibitor, *Hp*BARI, *H. polygyrus* Binds Alarmin Receptor and Inhibits, HSQC, ^1^H-^15^N shift correlation, HVL, hypervariable loop, ITC, isothermal titration calorimetry, SPR, surface plasmon resonance, TGF, transforming growth factor, TGM, transforming growth factor-β mimic

## Abstract

The mouse intestinal helminth *Heligmosomoides polygyrus* modulates host immune responses by secreting a transforming growth factor (TGF)-β mimic (TGM), to expand the population of Foxp3^+^ T_regs_. TGM comprises five complement control protein (CCP)-like domains, designated D1-D5. Though lacking homology to TGF-β, TGM binds directly to the TGF-β receptors TβRI and TβRII and stimulates the differentiation of naïve T-cells into T_regs_. However, the molecular determinants of binding are unclear. Here, we used surface plasmon resonance, isothermal calorimetry, NMR spectroscopy, and mutagenesis to investigate how TGM binds the TGF-β receptors. We demonstrate that binding is modular, with D1-D2 binding to TβRI and D3 binding to TβRII. D1-D2 and D3 were further shown to compete with TGF-β(TβRII)_2_ and TGF-β for binding to TβRI and TβRII, respectively. The solution structure of TGM-D3 revealed that TGM adopts a CCP-like fold but is also modified to allow the C-terminal strand to diverge, leading to an expansion of the domain and opening potential interaction surfaces. TGM-D3 also incorporates a long structurally ordered hypervariable loop, adding further potential interaction sites. Through NMR shift perturbations and binding studies of TGM-D3 and TβRII variants, TGM-D3 was shown to occupy the same site of TβRII as bound by TGF-β using both a novel interaction surface and the hypervariable loop. These results, together with the identification of other secreted CCP-like proteins with immunomodulatory activity in *H. polygyrus*, suggest that TGM is part of a larger family of evolutionarily plastic parasite effector molecules that mediate novel interactions with their host.

Helminth parasites are major human and animal health burdens in tropical regions of the world, with up to two billion infected humans worldwide (1, 2). The widespread association of helminths with mammals, together with the diversity of their lifecycles and niches, reflects an evolutionarily refined ability to manipulate the immune system using multiple molecular strategies (3, 4, 5). Helminth infections are often associated with an upregulation of regulatory T cells (T_regs_), either through expansion of the host’s pre-existing T_regs_ or by inducing *de novo* differentiation of peripheral T cells into the T_reg_ subset (6, 7, 8). T_regs_ potently promote immune tolerance by suppressing effector cell function (9), and in parasite-infected animals, they can restrict antiparasite immunity. Infection of mice with the intestinal helminth *Heligmosomoides polygyrus* increases the population of T_regs_, and worm clearance can be induced by antibody-mediated depletion of T_regs_ (10). In a physiologic context, the pleiotropic cytokine transforming growth factor (TGF)-β can induce differentiation of naïve T cells into T_regs_ through the defining transcription factor Foxp3 (11, 12, 13). In accord with this, we demonstrated that *H. polygyrus* excretory-secretory products (HESs) stimulate the differentiation of naïve T cells into T_regs_ by signaling through the TGF-β receptors, TβRI and TβRII (14). In recent studies, the protein in HES responsible for stimulating the TGF-β pathway and inducing T_regs_ was identified as a secreted five-domain 420-amino acid protein, designated as TGF-β mimic, or transforming growth factor-β mimic (TGM) (15). TGM induces signaling in both murine (16) and human (17) T cells with an efficacy comparable to TGF-β itself and binds directly to the host TGF-β receptors, TβRI and TβRII, despite bearing no sequence similarity to TGF-β, or any other member of the TGF-β family (15).

TGF-β homodimers are comprised of two 112-amino acid cystine-knotted monomers tethered together by a single interchain disulfide bond. They signal by assembling a heterotetrameric complex with two pairs of two serine/threonine kinase receptors, known as the TGF-β type I and type II receptors, TβRI and TβRII (18, 19, 20). The three TGF-β isoforms, TGF-β1, TGF-β2, and TGF-β3, control a multitude of pathways in cellular differentiation (21, 22, 23) and immune homeostasis (12, 21, 24), and TGF-β-dependent differentiation of naïve CD4^+^ cells into CD4^+^ CD25^+^ Foxp3^+^ T_regs_ is essential for peripheral immune tolerance (11, 12). Mice lacking TGF-β1, which is expressed by most cells and tissues, exhibit perinatal mortality and develop multiorgan inflammatory disease and die after maternal TGF-β1 is depleted (21). The dysregulation of the TGF-β pathway has been implicated in the pathogenesis of several human diseases, including inflammatory bowel disease (25), renal and cardiac fibrosis (26, 27), and soft-tissue cancers (26, 28, 29). In the latter setting, TGF-β drives immune exclusion, which promotes cancer progression and can prevent effective checkpoint therapy (30, 31). Thus, TGF-β is a key therapeutic target in its own right (32, 33).

TGM, in contrast to the single-domain structure of TGF-β, is composed of five modular domains, designated D1 – D5, all with distant sequence homology to proteins of the complement control protein (CCP) family (15). CCP domains are approximately 60 to 65 amino acids in length with multiple short β-strands tethered together by two highly conserved disulfide bonds in a Cys^I^-Cys^III^ and Cys^II^-Cys^IV^ topology (34). They are usually found in arrays and are present in numerous proteins, including the family of proteins that regulate complement, such as decay accelerating factor, factor H, and complement C3b/C4b receptor 1 (CR1) (34). In *H. polygyrus*, more than 30 CCP-containing proteins have been identified (35, 36), including in addition to TGM and nine TGM homologs (35), *H. polygyrus* Alarmin Release Inhibitor (*Hp*ARI) and *H. polygyrus* Binds Alarmin Receptor and Inhibits (*Hp*BARI), which suppress innate and adaptive type II immune responses, by binding IL-33 and its receptor ST2, respectively (37, 38, 39). Similar to TGM, *Hp*ARI and *Hp*BARI contain multiple CCP domains (three and two, respectively) and contain large insertions not present in canonical CCP domains (15, 37, 38).

Here, we characterized the individual domains of TGM and investigated the nature of the TGM:TβRI and TGM:TβRII binding interactions, using surface plasmon resonance (SPR), isothermal titration calorimetry (ITC), and NMR. Binding of TGM to the TGF-β receptors was found to be modular in nature, with D1-D2 and D3 binding TβRI and TβRII, respectively. TGM was additionally shown to bind to similar structural motifs on TβRI and TβRII as TGF-β, indicating that TGM truly mimics TGF-β, despite its lack of structural similarity. The solution structure of TGM-D3 was determined and showed that TGM-D3 assumes the overall fold of a CCP domain with two key differences: (1) a loop and a short helix replace two β-strands and (2) a long (23-amino acid) structurally ordered insertion within the hypervariable loop (HVL). These modifications lead to a significant lateral expansion of the domain and create potential interaction surfaces on opposite faces of the protein. Through NMR binding studies, as well as binding studies of TGM-D3 and TβRII variants, TGM-D3 is shown to engage TβRII through one of its two potential interaction surfaces, as well as through the HVL. These new structural data illuminate how *H. polygyrus* has adapted its own CCP domain–containing proteins for the purpose of protein mimicry and host immunomodulation.

## Results

### TGM binds to TβRI and TβRII using D1-D2 and D3, respectively

Previous *in vitro* TGF-β bioassays demonstrated that only TGM domains 1 to 3 were required for induction of CD4^+^ CD25^+^ Foxp3^+^ T_regs_ from naïve murine T cells or activation of a TGF-β reporter in a mouse embryonic fibroblast cell line (35). Proteins lacking domains 4 and 5 (TGM-D123) retained ability to induce TGF-β signaling, albeit with reduced potency in T-cell assays, while removal of any or all of domains 1 to 3 completely abolished activity. TGM was furthermore shown to require both TβRI and TβRII to elicit TGF-β signaling (15), as TGM activity was inhibited by both SB431542, a TβRI kinase inhibitor (40), and ITD-1, which stimulates ubiquitin-dependent degradation of TβRII (41). Previous SPR measurements demonstrated that TGM binds TβRII with micromolar affinity, similar but weaker than TGF-β1 and -β3, but unlike TGF-β1 and TGF-β3, which only bind TβRI with low nanomolar affinity once bound to TβRII, TGM binds TβRI with low nanomolar affinity in the absence of TβRII (14).

**Figure 1:**
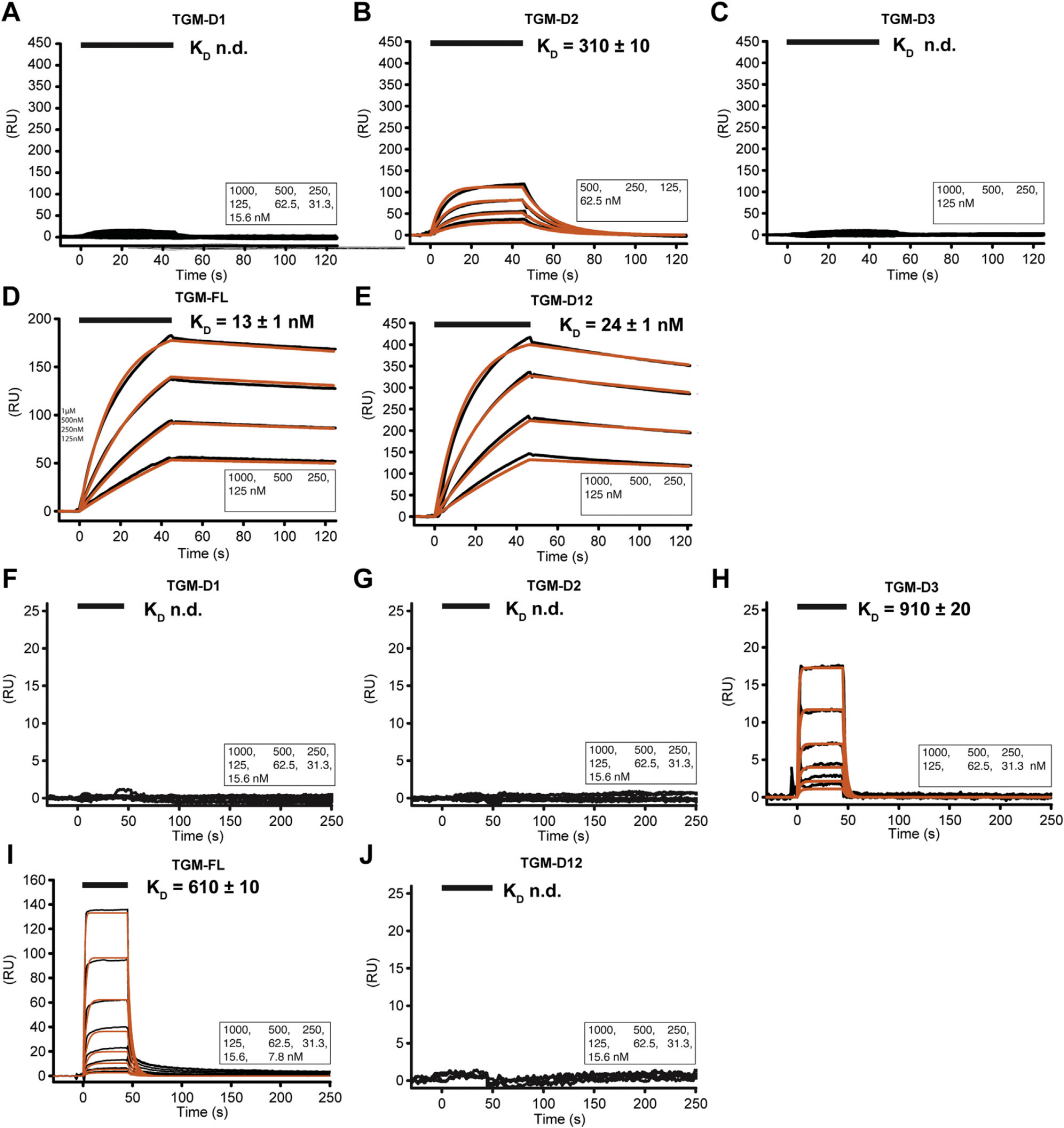
**Binding of TβRI and TβRII by TGM-D1, TGM-D2, TGM-D3, TGM-D12, and TGM-FL as assessed by SPR.***A–J*, SPR sensorgrams obtained upon injection of TGM-D1 (*A*, *F*), TGM-D2 (*B*, *G*), TGM-D3 (*C* and *H*), TGM-FL (*D* and *I*), or TGM-D12 (*E* and *J*) over immobilized TβRI (*A*–*E*) or TβRII (*F*–*J*). Sensorgrams, obtained upon injections of a 2-fold dilution series of each TGM construct, are shown in *black*, with the fitted curves in *orange* (data for TGM-D1:TβRI, TGM-D3:TβRI, TGM-D1:TβRII, TGM-D2:TβRII, and TGM-D12:TβRII were not fit due to weak signal). *Black bars* shown above the sensorgrams specify the injection period. Injected concentrations are shown in the lower right. SPR, surface plasmon resonance.

It is unknown which domains of TGM bind to TβRI and TβRII or if TβRI and TβRII directly contact one another, as in the TGF-β receptor complex. To investigate this, the individual domains TGM-D1, TGM-D2, and TGM-D3, along with full-length TGM (TGM-FL), were expressed and purified for SPR binding studies with the TGF-β receptors. The injection of these domains over biotinylated avi-tagged TβRI captured on a streptavidin-coated sensor chip yielded robust concentration-dependent responses when TGM-D2 or TGM-FL was injected, but not when TGM-D1 or TGM-D3 was injected (Fig. 1, *A*–*D*). The K_D_ values derived by globally fitting the TGM-D2 and TGM-FL sensorgrams to a (1:1) kinetic model were 310 nM and 13 nM, respectively (Table 1). Thus, TGM-D2 is evidently the main binding partner for TβRI, but nonetheless lacks the full binding capacity of TGM. The same series of injections, performed over biotinylated avi-tagged TβRII captured on a streptavidin-coated sensor chip, yielded robust responses when TGM-D3 or TGM-FL was injected, but not when TGM-D1 or TGM-D2 was injected (Fig. 1,*F*–*I*). The K_D_ values derived from the TGM-FL and TGM-D3 sensorgrams were 610 nM and 910 nM, respectively (Table 1). Thus, TGM-D3 accounts for most of the binding affinity of TGM-FL for TβRII.

**Table 1:** TGM:TβRI and TGM:TβRII binding as assessed by SPR

Surface	Analyte	Fitted parameters^a^			
		*k*_on_ (M^−1^ s^−1^)	*k*_off_ (s^−1^)	*K*D (nM)	*R*_max_ (RU)
TβRI	TGM-D1	ND^b^	ND^b^	ND^b^	ND^a^
TβRI	TGM-D2	(3.0 ± 0.1) × 10^5^	(9.1 ± 0.1) × 10^−2^	310 ± 10	89.6 ± 0.7
TβRI	TGM-D3	ND^b^	ND^b^	ND^b^	ND^a^
TβRI	TGM-D12	(6.7 ± 0.1) × 10^4^	(1.6 ± 0.1) × 10^−3^	24 ± 1	429 ± 1
TβRI^c^	TGM-FL	(5.9 ± 0.1) × 10^4^	(7.8 ± 0.2) × 10^−4^	13 ± 1	193 ± 1
TβRII	TGM D1	ND^b^	ND^b^	ND^b^	ND^a^
TβRII	TGM D2	ND^b^	ND^b^	ND^b^	ND^a^
TβRII	TGM D1D2	ND^b^	ND^b^	ND^b^	ND^a^
TβRII	TGM D3	(6 ± 1) × 10^5^	0.6 ± 0.1	910 ± 20	33.0 ± 0.4
TβRII	TGM FL	(2 ± 6) × 10^7^	(1 ± 4) × 10^−1^	610 ± 10	215 ± 2

a Fitted parameters were derived from kinetic analysis of a single injection series.

b Not determined due to weak signal.

c Measured on a lower density chip compared to that used for TβRI:TGM-D2 and TβRI:TGM-D12.

TGM-D3’s full and TGM-D2’s partial recapitulation of TGM binding affinity for TβRII and TβRI, respectively, suggested that TGM-D1 might contribute to binding of TβRI. Thus, we assessed binding of a construct containing both TGM-D1 and TGM-D2, designated TGM-D12, to TβRI and TβRII using SPR. This didomain construct bound robustly to TβRI, but did not bind at all to TβRII (Fig. 1, *E* and *J*). The K_D_ derived from kinetic analysis of the TGM-D12:TβRI sensorgrams was 24 nM, which is within a factor of two of that of TGM-FL (Table 1). Thus, TGM-D1 also contributes to the binding to TβRI.

**Figure 2:**
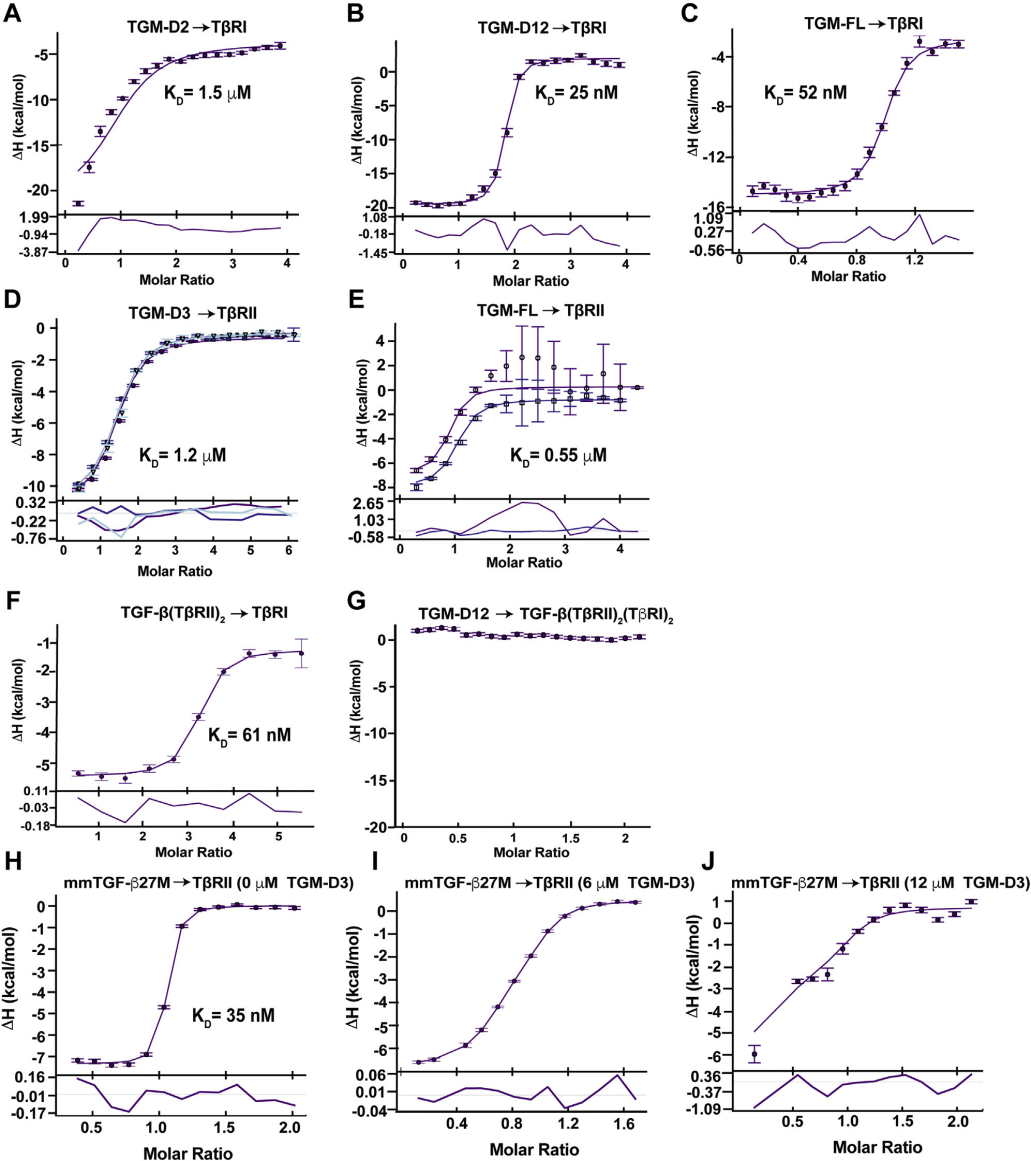
**TGM binding to TβRI and TβRII and competition with TGF-β by ITC.***A–E*, integrated heats for the injection of TGM-D2 (*A*), TGM-D12 (*B*), or TGM-FL (*C*) into TβRI, and TGM-D3 (*D*) or TGM-FL (*E*) into TβRII, together with the fit (*smooth line*) and residuals (below) to a 1:1 binding model. Error bars indicate bias in the NITPIC estimation of the integrated heats. *F* and *G*, TGM-D12 and TGF-β(TβRII)_2_ competitive binding to TβRI. Integrated heats obtained upon injection of TGF-β(TβRII)_2_ (*F*) into TβRI or TGM-D12 into TβRI with a saturating concentration of TGF-β(TβRII)_2_ binary complex (*G*). *H–J*, TGM-D3 and mmTGF-β27Μ competitive binding to TβRII. Integrated heat obtained upon injection of 150 μM mmTGF-β27M into 15 μM TβRII in the sample cell with 0 μM (*H*), 6.0 μM (*I*), or 12.0 μM (*J*) TGM-D3. The data points correspond to the integrated heats and the *smooth lines* a global fit over the three datasets to a 1:1 binding model with competition. ITC, isothermal titration calorimetry.

ITC experiments, which in contrast to SPR are carried out entirely in solution and do not require any tagging, were also performed to assess binding of the individual domains of TGM to TβRI and TβRII. In accord with the SPR results, titration of TGM-D2, TGM-D12, and TGM-FL into TβRI and TGM-D3 and TGM-FL into TβRII yielded readily measurable binding isotherms with large negative enthalpies (Figs. 2, *A*–*E* and S1, *A*–*E*). In contrast, titration of TGM-D1 and TGM-D3 over a similar range of concentrations into TβRI and TGM-D1 and TGM-D2 into TβRII did not (Fig. S1, *F*–*M*). In further accord with the SPR results, the fitted K_D_ values for binding of TGM-D12 to TβRI and TGM-D3 to TβRII were comparable to those of TGM-FL and were generally consistent with those measured by SPR (Table S2). In contrast, and as expected based on the SPR results, the K_D_ for binding of TGM-D2 to TβRI was significantly increased (ca. 50-fold) relative to TGM-FL (Table S2). ITC, in addition to providing K_D_ values, also provides values for the stoichiometry, and as shown, TGM-FL binds both TβRI and TβRII with near 1:1 stoichiometry (Table S2). The near 1:1 stoichiometry is also observed for the TGM subdomains shown to bind TβRI and TβRII, TGM-D12 and TGM-D3, respectively, but for TGM-D2 binding to TβRI, the stoichiometry was closer to 0.5. The differing stoichiometry for binding of TGM-D2 and TGM-D12 to TβRI is likely due the weaker affinity of the former interaction, which makes accurate data fitting difficult. Thus, as discussed in a following section, an alternative method was used and this established 1:1 stoichiometry for the TGM-D2 to TβRI interaction.

### TGM competes with TGF-β for binding to TβRI and TβRII

To assess potential shared binding sites on TβRI and TβRII, ITC competition experiments were performed in which K_D_s and enthalpies for TβRI and TβRII binding to their partners were measured under noncompetitive and competitive conditions. In the case of TβRI, titration of the TGF-β3(TβRII)_2_ complex into TβRI yielded a fitted K_D_ of 61 nM (Fig. 2*F* and Table S3), which is similar to the K_D_ of 25 nM when TGM-D12 was titrated into TβRI (Fig. 2*B* and Table S2). However, unlike TGM-D12:TβRI binding which had a large negative enthalpy, −19 kcal mol^−1^ (Table S2), binding of TGF-β3(TβRII)_2_ to TβRI had a much smaller negative enthalpy, −4.2 kcal mol^−1^, even at an increased temperature (Table S3). In light of similar K_D_s, but significantly different enthalpies, the competition experiment with TβRI was performed by titrating TGM-D12 into the cell loaded with the TGF-β3(TβRII)_2_(TβRI)_2_ ternary complex (Fig. 2*G*). This yielded no heat, indicating that TGM-D12 and TGF-β3(TβRII)_2_ compete for binding to TβRI.

TGF-β3, and TGF-β homodimers in general, is well known to be practically insoluble in the unbound form, except under either very acidic (pH 4.0) or basic (pH 11.0) conditions (42). Therefore, competition experiments in which TβRII is titrated into TGM (or TGM-D3), together with TGF-β3 as a competitor, are not feasible. To circumvent this, mmTGF-β27M, an engineered TGF-β monomer, which is soluble at neutral pH and binds TβRII in a manner indistinguishable from TGF-β3, was used (43). In the absence of competitor, titration of mmTGF-β27M into TβRII yielded a large negative enthalpy and a sharp binding transition, consistent with a low nanomolar binding affinity as previously reported (Fig. 2*H*) (43). In the presence of increasing concentrations of competitor, either 6 or 12 μM TGM-D3, there was a readily detectable increase in the curvature, consistent with competitive binding (Fig. 2,*I* and *J*). In order to analyze the data, the integrated heats from the three experiments, together with fitted K_D_ and enthalpy for the TGM-D3:TβRII interaction (Table S2), were globally fit to a simple competitive binding model to derive the K_D_ for high affinity mmTGF-β27M:TβRII binding (Fig. 2, *H*–*J* and Table S3). The K_D_ was found to be 35 nM, in accord with previous SPR measurements for the TβRII:TGF-β interaction with immobilized TGF-β1 or TGF-β3 (43). This demonstrates that TGM-D3 and mmTGF-β27M, and by logical extension TGM and TGF-β homodimers, compete for binding to TβRII.

### TGM binds TβRI with high affinity due to direct binding of both TGM-D1 and TGM-D2

The SPR and ITC experiments have shown that TGM-D12 recapitulates nearly the full-binding affinity of TGM-FL to TβRI, while TGM-D2 alone is 20 to 50 fold weaker. In spite of the apparent contribution of TGM-D1, its direct binding to TβRI was not detected using either SPR or ITC. In order to investigate the possibility that TGM-D1 does directly bind TβRI, but too weakly to be detected within the range of affinities possible by SPR or ITC, we prepared ^15^N-labeled TGM-D1, as well as ^15^N-TGM-D2 and ^15^N-TGM-D3, and examined binding to TβRI using NMR spectroscopy.

The two-dimensional (2D) ^1^H-^15^N shift correlation (HSQC) spectra of both TGM-D2 and TGM-D3 were both well dispersed, with numerous peaks outside of the random coil limit (7.8–8.5 ppm in the ^1^H dimension), demonstrating that these proteins are natively folded (Fig. S2, *A* and *C*). The number of backbone amide signals for TGM-D3 was close to the number expected (77 observed, 81 expected), while for TGM-D2, the total number of signals exceeded that expected (106 observed, 76 expected). To determine if the additional signals in TGM-D2 were due to sample heterogeneity, for example, as a result of slow conformational dynamics, HSQC ZZ-exchange spectra with mixing times ranging between 0 to 250 ms were recorded (44). These experiments identified at least 12 pairs of peaks undergoing exchange on this timescale, indicating that the protein is undergoing a slow conformational transition that leads to two forms in solution (Fig. S2, *A* and *B*). The process responsible was not investigated but might be proline *cis*:*trans* isomerization, as this is known to occur on slow timescales (45) and TGM-D2 has four additional proline residues relative to TGM-D3 (Table S1).

TGM-D1, in contrast to TGM-D2 and TGM-D3, had poor signal dispersion, with most peaks clustered in the random coil region of the spectrum (Fig. S3*A*). To investigate the possibility that TGM-D1 was natively folded, but aggregated, CHAPS in increasing concentrations was added to the buffer and the protein concentration was decreased. This led to the appearance of a large number of peaks outside of the random coil region (Fig. S3, *B*–*D*). The spectrum with 20 μM TGM-D1 and 10 mM CHAPS in the buffer had roughly the expected number of peaks (46) but also a few intense peaks in the random coil region of the spectrum. Thus, TGM-D1 appears to be natively folded, but perhaps still partially aggregated under these conditions.

**Figure 3:**
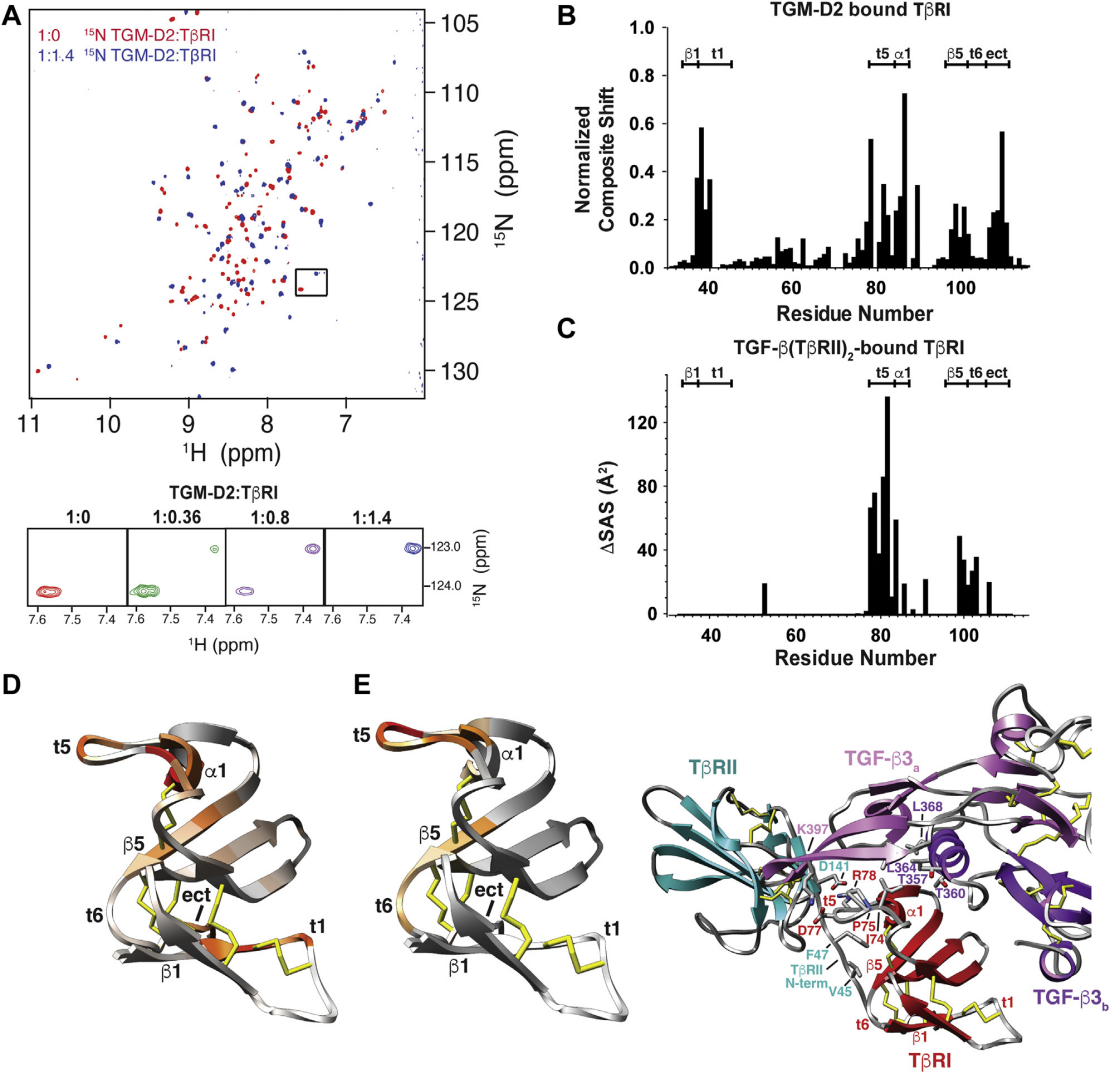
**Binding of TGM-D2 to TβRI.***A*, ^1^H-^15^N HSQC spectra of 0.2 mM ^15^N TGM-D2 alone (*red*) overlaid with the spectrum of the same sample, but with 1.2 M equivalents of unlabeled TβRI added (*blue*). Spectra were recorded in 25 mM sodium phosphate, 50 mM sodium chloride, and 5% ^2^H_2_O, pH 7.0, at 310 K. Expansion of the boxed region with intermediate titration points is shown below. *B* and *D*, plot of the composite shift perturbations of TβRI upon binding to TGM-D2 (*B*) and a depiction of these on the structure of TβRI from PDB 2PJY (*D*). Structure is colored using a scale where white indicates minimal composite shift perturbation and red indicates maximal. *C* and *E*, plot of the difference in solvent-accessible surface area for individual residues of TβRI between the free and bound form (PDB 2PJY) (*C*) and a depiction of these on the structure of TβRI from PDB 2PJY (*E*, *left*). Structure is colored using a scale where *gray* indicates minimal ΔSAS and red indicates maximal ΔSAS. Shown also in (*E*) (*right*) is the structure of one side of the TGF-β3(TβRII)_2_(TβRI)_2_ complex, with the two monomers of TGF-β3 depicted in pink and magenta, TβRII in *cyan*, and TβRI in *red*. Key residues at the interface between TGF-β3:TβRII and TβRI are shown. HSQC, ^1^H-^15^N shift correlation.

To assess binding, ^15^N-labeled TGM-D1, TGM-D2, or TGM-D3 was combined with increasing amounts of unlabeled TβRI, ranging from 0 to 1.4 equivalents. This resulted in significant perturbations in the backbone amide signals of TGM-D2 (Fig. 3*A*), but not those of either TGM-D1 or TGM-D3 (Fig. S4, *A* and *B*), consistent with the SPR and ITC results. The signals of ^15^N-TGM-D2 underwent slow-exchange conversion from the free to the bound form as increasing amounts of TβRI were added, but were not fully converted to the bound form until more than 0.8 equivalents of TβRI were added (Fig. 3*A*). Thus, TGM-D2 appears to bind TβRI with 1:1 stoichiometry, not 0.5:1 as suggested by the ITC titration. The binding of TβRI was further shown to resolve the conformational doubling apparent in TGM-D2 (Fig. S4, *C* and *D*), indicating that binding stabilizes TGM-D2 in one of its two forms.

The spectrum of ^15^N TGM-D1 with 1.4 equivalents of unlabeled TβRI added was recorded with 10 mM CHAPS in the buffer. This might impede binding, and thus, a role of TGM-D1 in binding TβRI cannot be excluded. Thus, the converse experiment was performed, with ^15^N-labeled TβRI combined with 1.2 M equivalents of unlabeled TGM-D1, TGM-D2, or TGM-D3, all in buffers lacking CHAPS. The addition of TGM-D2 caused large perturbations in most of the signals of TβRI, whereas addition of TGM-D3 led to no perturbations, consistent with the inverse experiments (Fig. S5, *A* and *B*). The addition of TGM-D1 in the absence of CHAPS resulted in the weakening or full disappearance of most of the TβRI backbone signals, along with small chemical shift perturbations of other signals (Fig. S5*C*). The disappearance of these signals is likely due to ^15^N-TβRI binding TGM-D1 and being incorporated into a TGM-D1 aggregate. Thus, TGM-D1 does appear to bind TβRI and the high affinity of TGM-FL for TβRI is likely a result of multivalent binding, in which TGM-D1 and TGM-D2 both directly bind TβRI.

### TGM-D2 and TGF-β:TβRII bind a similar set of residues on TβRI

The ITC competition experiments clearly demonstrated that TGM-D12 and TGF-β3(TβRII)_2_ complex compete with one another for binding TβRI, suggesting that TGM-D12 recognizes and binds a set of residues that partially or fully overlap with that bound by TGF-β3(TβRII)_2_. To further investigate, we prepared a sample of ^13^C,^15^N TβRI bound to a slight excess of unlabeled TGM-D2 (as the complex with TGM-D12 proved to be intractable) and assigned the backbone HN, N, Cα, C^O^, and Cβ resonances for all nonproline residues, except for Cys^41^-Thr^42^, Ser^69^-Cys^71^, Ala^87^, and Ser^90^-Thr^92^ (Fig. S6*B*). To identify potential interface residues, the assigned chemical shifts for TGM-D2-bound TβRI were compared to those previously reported for unbound TβRI under similar buffer conditions (Figs. 3*B* and S6*A*) (18). The largest chemical shift perturbations (CSPs) fell within three regions. The first is the C-terminal end of β1 and the turn that follows (t1), amino acids 32 to 40 (Fig. 3, *B* and *D*). This region of TβRI does not interact at all with TGF-β(TβRII)_2_ (Fig. 3, *C* and *E*). The second, turn 5 (t5), also known as the Pro-Arg-Asp-Arg-Pro (PRDRP) prehelix extension, and the short 1 turn helix that follows (α1) (Fig. 3, *B* and *D*), is the contact between TGF-β and TβRII and residues 78 to 87 of TβRI (Fig. 3, *C* and *E*), while the third is β-strand 5 and the following extended C-terminus (Fig. 3, *B* and *D*) which is the interface between the structurally ordered N-terminal tail of TβRII and residues 97 to 110 of TβRI (Fig. 3, *C* and *E*). Thus, one domain of TGM, D2, has evolved not only to replicate the binding properties of two host proteins (TGFβ and TβRII) but also to form a third novel site that may confer its overall higher affinity for the receptor.

### TGM-D3 and TGF-β bind a similar set of residues on TβRII

**Figure 4:**
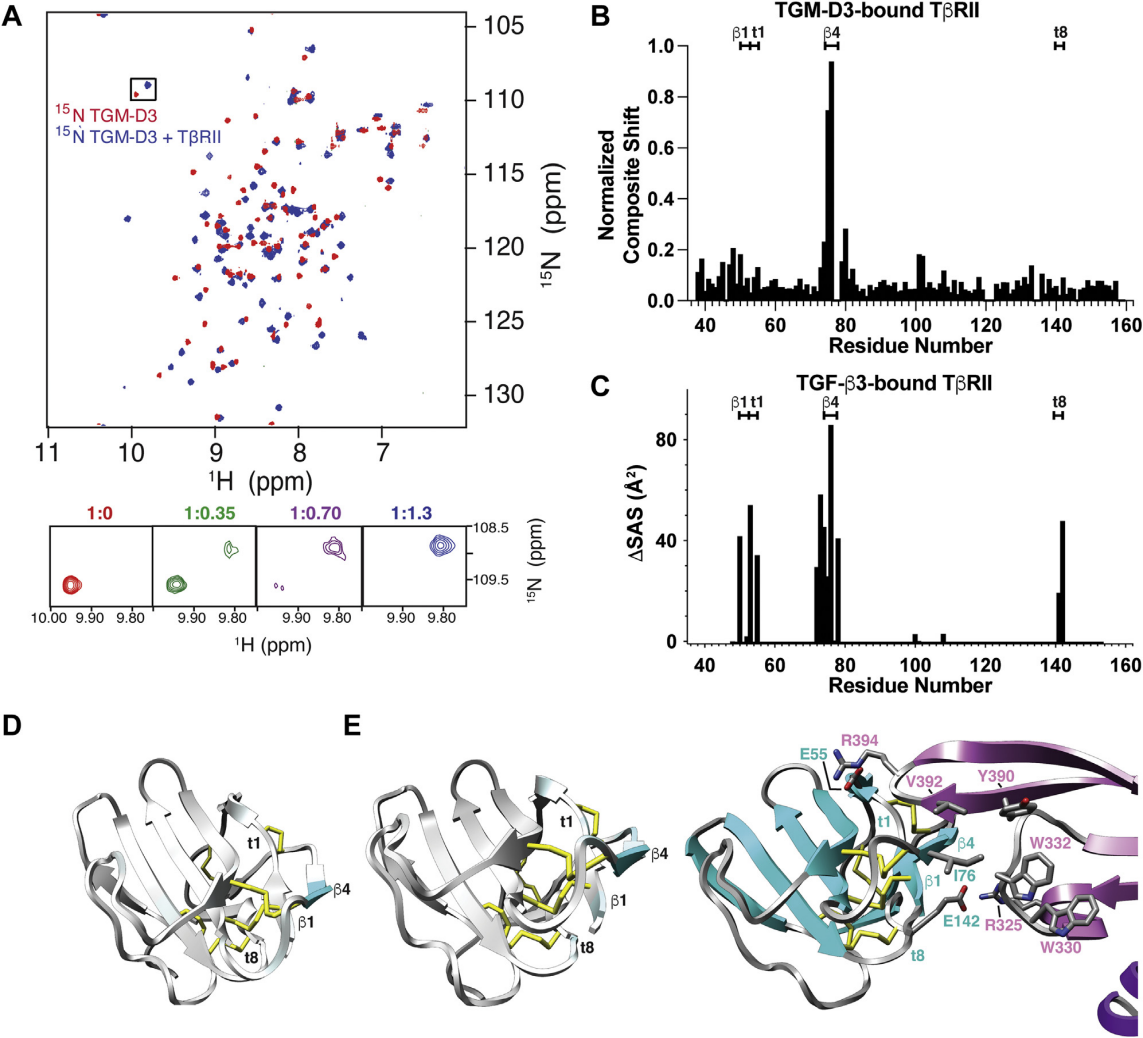
**Binding of TGM-D3 to TβRII.***A*, ^1^H-^15^N HSQC spectra of 0.2 mM ^15^N TGM-D3 alone (*red*) overlaid with the spectrum of the same sample, but with 1.2 M equivalents of unlabeled TβRII added (*blue*). Spectra were recorded in 25 mM sodium phosphate, 50 mM sodium chloride, and 5% ^2^H_2_O, pH 6.0, at 310 K. Expansion of the boxed region with intermediate titration points is shown below. *B* and *D*, plot of the composite shift perturbations of TβRII upon binding to TGM-D3 (*B*) and a depiction of these on the structure of TβRII from PDB 1KTZ (*D*). Structure is colored using a scale where *white* indicates minimal composite shift perturbation and *cyan* indicates maximal. *C* and *E*, plot of the difference in solvent accessible surface area for individual residues of TβRII between the free and bound form (PDB 1KTZ) (*C*) and a depiction of these on the structure of TβRI from PDB 2PJY (*E*, *left*). Structure is colored using a scale where *gray* indicates minimal ΔSAS and *cyan* indicates maximal ΔSAS. Shown also in panel *E* (*right*) is the structure of one side of the TGF-β3(TβRII)_2_ complex, with the two monomers of TGF-β3 depicted in *pink* and *magenta* and TβRII in *cyan*. Key residues at the interface between TGF-β3 and TβRII are shown. HSQC, ^1^H-^15^N shift correlation.

The binding of ^15^N TGM-D1, TGM-D2, and TGM-D3 by unlabeled TβRII and ^15^N TβRII by unlabeled TGM-D1, TGM-D2, and TGM-D3 was also investigated using NMR. This revealed multiple perturbations in ^15^N TGM-D3 signals, but none with ^15^N TGM-D1 or ^15^N TGM-D2 when unlabeled TβRII was added (Figs. 4*A* and S7, *A* and *B*); similarly, many of the signals of ^15^N TβRII were perturbed by TGM-D3, but not by TGM-D1 or TGM-D2 (Fig. S7, *C*–*E*). These results, in addition to being internally consistent, also conformed to the overall conclusions derived from the earlier analyses by SPR and ITC.

To identify specific residues of TβRII that are recognized and bound by TGM-D3, the backbone signals of ^15^N,^13^C TβRII bound to unlabeled TGM-D3 were assigned (Fig. S8*B*) and compared to those previously reported for the unbound form (Fig. S8*A*) (47). The largest chemical shift perturbations, as deduced from a composite of the HN, N, Cα, Cβ, and C^O^ resonances, fell within a narrow region from residues 75 to 77 (Fig. 4, *B* and *D*). This region corresponds closely with the primary region of TβRII that binds TGF-β (Fig. 4, *C* and *E* left), demonstrating that the same motif of TβRII, the β4 edge strand that binds deeply in the cleft between the fingers 1 to 2 and 3 to 4 (47, 48) of TGF-β, is also engaged by TGM-D3. The binding of TGM-D3 leads to only minor shift perturbations outside of TβRII β4 (Fig. 4*B*), whereas TGF-β3 also directly engages residues 50 to 55 and 141 to 142 (Fig. 4, *C* and *E* right). Thus, while these might still be contacted by TGM-D3, as suggested by small composite shift perturbations in these regions (Fig. 4*B*), it appears that these contacts may not be as intimate as those with TGF-β.

### TGM-D3 structure and dynamics

**Figure 5:**
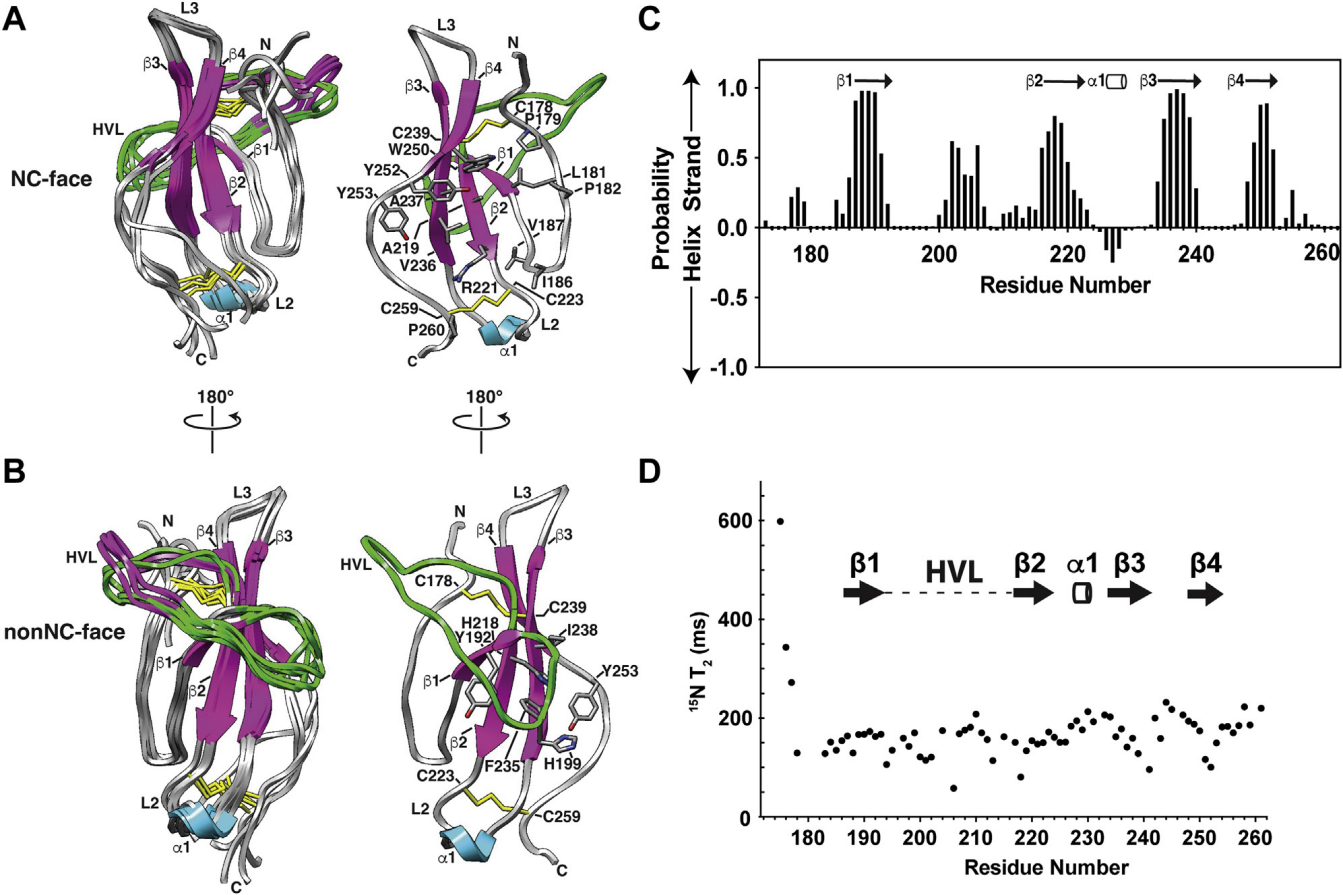
**Structure and backbone dynamics of TGM-D3.***A* and *B*, shown on the *left* are an ensemble of the five lowest-energy NMR structures of the unbound form of TGM-D3: β-strands, *magenta*; loops, *gray*; 3_10_ helix, *cyan*; disulfide bonds, *yellow*, two conformations of HVL highlighted in *green* and *pink*. Key structural features are indicated. Orientations shown differ by a 180-degree rotation around the y-axis, with orientation shown in (*A*) highlighting the face of the protein that includes N- and C-terminus (NC-face) and the orientation shown in (*B*) highlighting the opposite face (non-NC-face). Shown on the *right* are single representative structures, with the four cysteines that form the two disulfide bonds and the side chains of key residues highlighted. *C*, PECAN-based prediction of TGM-D3 secondary structure. Positive values indicate β-strand probability; negative values indicate helical probability. Spectra recorded in 25 mM sodium phosphate, 50 mM sodium chloride, and 5% ^2^H_2_O, pH 6.0, at 310 K. Secondary structure elements shown above correspond to those deduced from the calculated TGM-D3 solution structure. *D*, backbone ^15^N T_2_ relaxation times for TGM-D3 plotted per individual residue with structural features mapped. HVL, hypervariable loop.

The structure of TGM-D3 was determined based on near-complete chemical shift assignments for both the backbone and side chains, ^1^H-^1^H NOE distance restraints, ^1^H-^15^N, ^13^C^α^-^1^H^α^, and ^13^C^O^-^15^N RDCs, and ^3^J^HN-Hα^ J-couplings, with relevant statistics presented in Table S4. TGM-D3 is comprised of four β-strands (Tyr^189^-Gly^193^, Thr^217^-Arg^221^, Glu^234^-Lys^241^, and Ser^248^-Tyr^252^) arranged into a highly twisted antiparallel β-sheet with a β1:β2:β3:β4 topology (Fig. 5, *A* and *B*). The first β-strand is present in some but not all of the lowest-energy structures. There is also a 3_10_ helix (Gln^228^-Ala^230^) connecting β2 and β3 in some, but not all of the lowest-energy structures (Fig. 5, *A* and *B*). The structures are consistent with a PECAN analysis of secondary shifts (49), with four high probability extended regions predicted between residues 184 to 191, 216 to 222, 234 to 241, and 248 to 252, and a low probability helical region from residues 226 to 228 (Fig. 5*C*). The secondary shifts also predict, with lower probability, extended regions between residues 177 to 179 and 201 to 206. The former corresponds to the N-terminal region (Fig. 5*A*), while the latter corresponds to the middle section of the 23-residue HVL loop that connects β1 and β2 (Fig. 5*B*). This section of the HVL extends perpendicularly across the C-terminal end of β1 and packs on its N-terminal end against several bulky hydrophobic residues that emanate from the surface of the twisted sheet, including Tyr^192^, His^218^, Ile^238^, and Phe^235^ (Fig. 5*B*). The HVL is mostly converged among the ten lowest energy structures, with an average backbone pairwise RMSD of 1.74 Å. The segments from residues 177 to 179 and 201 to 206, although highly extended, do not form hydrogen bonds that define a β-strand and thus are not classified as such in the calculated structures.

The Cys^178^-Cys^239^ disulfide pins the N-terminus to one end of the β-sheet, while the C-terminus is pinned to the other end by the Cys^223^-Cys^259^ disulfide (Fig. 5, *A* and *B*). The core of the protein is localized in the region circumscribed by the extended N-terminal segment on one side and β4 and the extended segment that follows on the other side (Fig. 5*A*). The hydrophobic residues in the core include Leu^181^ and Pro^182^ from the extended N-terminal segment, Ile^186^, Val^187^, and Tyr1^89^ from β1, Ala^219^ and the hydrophobic portion of the side chain of Arg^221^ from β2, Val^236^ and Ala^237^ from β3, and Trp^250^ and Tyr^252^ from β4 (Fig. 5*A*).

The backbone ^15^N T_2_ relaxation times, which are sensitive to fast (ns-ps) timescale motions that result from low-amplitude fluctuations of the backbone, are significantly increased in the N-terminal tail and modestly increased near the C-terminal end of the HVL and in the shorter loops connecting β2-β3 and β3-β4 (Fig. 5*D*). The increases in ^15^N T_2_ indicate increased flexibility in these regions, especially the N-terminal tail which does not converge in the final ensemble of structures. The other loop regions converge reasonably well, consistent with their more modest increases in ^15^N T_2_ (Fig. 5*D*), although one exception is the HVL, which adopts two conformations, in which the C-terminal portion of the HVL either ascends or descends as it contacts the extended N-terminus (Fig. 5*B*, green and pink, respectively). There is a low percentage of Ramachandran outliers in the structure, but these are present in regions that are completely or partially unstructured, including the N- and C-termini and the C-terminal portion of the HVL.

### TGM-D3 is a remodeled CCP domain with a lateral expansion to expose hydrophobic sites

**Figure 6:**
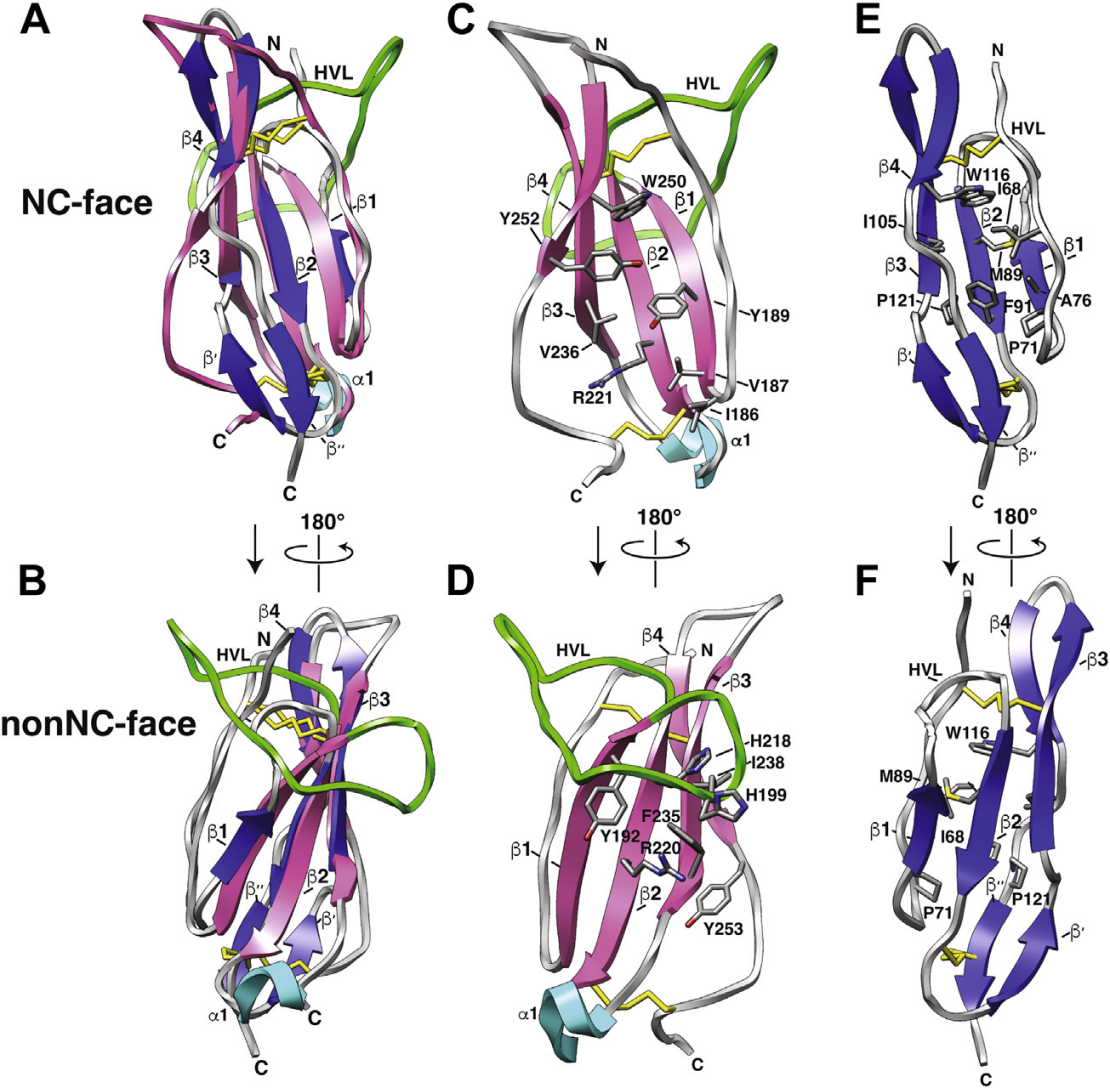
**TGM-D3 comparison to CCP domains.***A* and *B*, alignment of TGM-D3 to representative CCP domain, PDB 1CKL (*blue*), as viewed from the NC-face (*A*) or non-NC-face (*B*). TGM-D3: β-strands, *magenta*; loops, *gray*; 3_10_ helix, *cyan*, HVL, *green*; PDB 1CKL: β-strands, *blue*; loops, including HVL, *gray*. Key structural features are indicated. *C* and *D*, TGM-D3 in the same orientation as shown in *A* and *B*, respectively. Side chains of key residues on both the NC- and non-NC-faces are highlighted. *E* and *F,* PDB 1CKL in the same orientation as shown in *A* and *B*, respectively. Side chains of residues in the hydrophobic core are highlighted. CCP, complement control protein; HVL, hypervariable loop.

Structures with the closest similarity to TGM-D3, as identified by a DALI (50, 51) search of the Protein Data Bank, were all CCP-containing proteins, as anticipated based on previous bioinformatic analyses (15). Structural overlays show that the top hit, 1CKL (human CD46), as well as other top hits 2PSM (IL-15Rα), 1H2P (CD55), 5FO9 (CR1), and 5FOA (decay accelerating factor), have close correspondence of the four β-strands and the Cys^I^ -Cys^III^ and Cys^II^ -Cys^IV^ disulfides that form the core of the TGM-D3 fold (Fig. 6, *A* and *B*). However, in spite of the considerably longer length of TGM-D3 than that of the top-scoring CCP domains (90 and 65–75 residues, respectively), TGM-D3 lacks two short β-strands, one in the loop connecting β2 and β3, designated β′, and another at the C-terminus, designated β’’, present in all of the top-scoring CCP domains (Fig. 6, *C*–F, respectively). In conventional CCP domains, the pairing of the β′ and β’’ strands, together with the II-IV disulfide that bridges the β’’ strand to the C-terminal end of β2, serves to draw the C-terminal segment toward the loop connecting β2-β3, thereby creating a closed cavity that is packed with hydrophobic residues (Fig. 6, *E* and *F*). In TGM-D3, there is a significant lateral expansion of the domain due to the absence of the β′ and β’’ strands, which leads to a pronounced divergence of the extended segment that follows β4 away from the structurally ordered N-terminal segment (Fig. 6*D*). This lateral expansion is evident whether the protein is viewed from the face that includes the N- and C-terminus, designated as the NC-face (Fig. 6*C*), or the opposite face, designated as the non-NC-face, that includes the extended HVL (Fig. 6*D*). The expansion on the NC-face of the protein leaves several hydrophobic residues partly exposed to solvent, including Ile^186^, Val^187^, and Tyr^252^ (Fig. 6*C*). The expansion on the non-NC-face also leads to the partial exposure of several hydrophobic residues, including Tyr^253^ in the extended segment following β4 and Tyr^192^ and Phe^235^ which form part of the surface against which the HVL packs (Fig. 6*D*). Overall, the remodeling of TGM-D3 leads to a considerable lateral expansion of the domain and creates potential interaction surfaces on both the NC- and non-NC-faces for binding to TβRII.

### TGM-D3 engages its partner, TβRII, using structural motifs unique to TGM-D3

**Figure 7:**
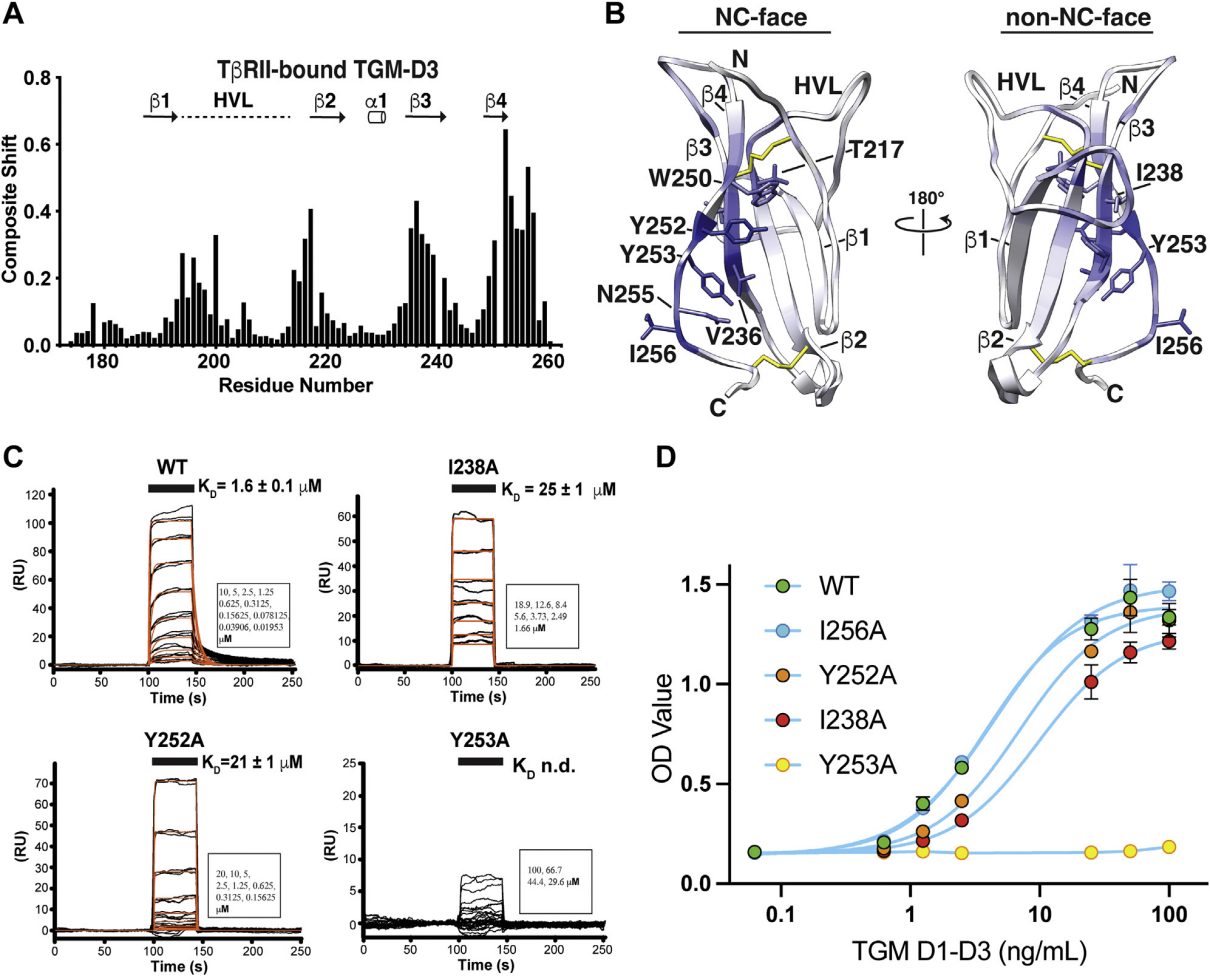
**Binding of TβRII to TGM-D3.***A* and *B*, composite shift perturbations of TGM-D3 upon binding to TβRII (*A*) and a depiction of these on the structure of TβRII (*B*). Secondary structure shown above the composite shifts in *A* corresponds to the secondary structure as deduced from the TGM-D3 solution structure. Structure in *B* is colored using a scale where white indicates minimal composite shift perturbation and *dark blue* indicates maximal shift perturbation. *C*, binding of TβRII by TGM-D3 variants as assessed by SPR. SPR sensorgrams obtained upon injection of WT, I238A, Y252A, or Y253A TGM-D3 over immobilized TβRII. Sensorgrams, obtained upon injection of a 2-fold dilution series of each TGM construct, are shown in *black*, with the fitted curves in *orange* (data for Y253A were not fit due to weak signal). *Black bars* shown above the sensorgrams specify the injection period. Injected concentrations are shown in the *lower right*. *D*, impact of TGM-D3 mutations on TGF-β signaling as measured through the MFB-F11 TGF-β responsive bioassay. I256A (*blue*), Y252A (*orange*), I238A (*red*), and Y253A (*yellow*) were assessed for TGF-β signaling and compared to WT TGM1-D13 (*green*). SPR, surface plasmon resonance.

To identify the binding interface on TGM-D3 for TβRII, the backbone of ^15^N,^13^C TGM-D3 was fully assigned as bound to unlabeled TβRII (Fig. S9*B*). This enabled differences in the assigned chemical shifts to be computed relative to the free form (Figs. 7*A* and S9*A*). These differences showed that the regions of TGM-D3 most strongly perturbed upon binding TβRII ranged from residues 234 to 243 and 249 to 257, which correspond to most of β3 and β4, as well as a few residues that extend beyond the end of β4 (Fig. 7, *A* and *B*). The regions perturbed to a lesser extent include residues 214 to 219 and 193 to 200, which correspond to the N-terminal end of β2 and the N-terminal end of the HVL. The residues maximally perturbed on β3 and β4 are present on the NC-face and non-NC-face of TGM-D3 and include Tyr^252^ and Val^236^ and Ile^238^, Tyr^253^, and Ile^256^, respectively (Fig. 7*B*).

To determine whether TβRII binds to the NC- or non-NC-face and to determine which residues contribute greatest to binding, we substituted residues of TGM-D3 within the NC- and non-NC-faces that could potentially interact with TβRII with alanine and assessed TβRII binding using SPR. The specific residues chosen for substitution included Val^236^, Tyr^252^, and Asn^255^ on the NC-face and Arg^198^, His^199^, Phe^235^, Ile^238^, Tyr^253^, and Ile^256^ on the non-NC-face. Lys^254^ and Lys^258^ in the extended region following β-strand 4 were also substituted. The variants were screened for native folding by recording the one-dimensional (1D) ^1^H NMR spectrum. Though some had small shifts in some of the resolved methyl and amide signals, all were found to be natively folded and none presented any evidence of gross folding abnormalities (Fig. S10).

The SPR response amplitudes were attenuated for several of the variants, including R198A, F235A, V236A, I238A, Y252A, and Y253A, indicating that the binding affinity was diminished (Figs. 7*C* and S11, *A*–*H*). The responses were nonetheless sufficient to obtain satisfactory fits for all of the variants, except Y253A, which was severely diminished (Figs. 7*C* and S11, *A*–*H*). The K_D_ values for the R198A, I238A, Y252A, and K254A variants were increased by more than 20-fold, while for the F235A and V236A variants, more modest increases of about 4-fold were observed (Table S5). The side chains of Arg^198^, Lys^254^, Tyr^253^, and Ile^238^ are all on the non-NC-face of the protein, while that of Tyr^252^, immediately adjacent to Tyr^253^, is located on the NC-face. The other residues located nearby Tyr^253^ that underwent large backbone CSPs, Asn^255^, and Ile^256^ resulted in little to no attenuation of binding upon substitution with alanine. Tentatively, this suggests the binding site for TβRII resides on the non-NC-face of the protein in the region that is formed by residues protruding from β3, the C-terminal end of β4, and the N-terminal end of the HVL. The large CSPs for residues with side chains on the NC-face of the protein is likely because their backbone atoms are contacted by TβRII, for example, Val^236^, Tyr^252^, and Asn^255^, or because of indirect transmission of binding-induced perturbations through the disulfide-stabilized structure, for example, Thr^217^, Trp^250^, and Ile^256^.

To assess the contribution of TβRII residues to binding, we performed a similar analysis in which we substituted Ile^73^, Ser^75^, and Ile^76^ in β4 with either alanine (Ile^73^ or Ile^76^) or leucine (Ser^75^). To investigate the possibility that residues of TβRII that formed hydrogen-bonded ion pairs with the fingertip regions of TGF-β, Asp^55^, and Glu^142^, also interact with TGM-D3, we also substituted these with asparagine and glutamine, respectively. The SPR measurements showed that all variants within β4 perturbed binding. The S75L variant in the center of the strand increased the K_D_ by nearly 200-fold, whereas the I76A and I73A variants increased the K_D_ by 26- and 7-fold, respectively (Figs S11, *E*–H and Table S5). The variants at flanking positions also significantly increased the K_D_, with D55N and E142Q variants increasing the K_D_ by 63- and 17-fold, respectively (Figs S11, *I*–*J* and Table S5). Thus, in spite of the modest binding-induced CSPs at these positions, these residues nonetheless contribute significantly to binding. These residues may interact with TGM-D3 Arg^198^ and Lys^254^, which when mutated to alanine increased the K_D_ for binding TβRII by over 30-fold (Figs. S11 and Table S5). Thus, TGM-D3 appears to closely mimic TGF-β by engaging TβRII not only through β4 but also by interacting with Asp^55^ and Glu^142^ which flank β4 in the structure of TβRII (Fig. 4*E*, right).

To ascertain if the residues in TGM-D3 important for TβRII binding were also functionally important, we evaluated the effect of four representative substitutions on signaling activity using the highly sensitive MFB-F11 TGF-β reporter bioassay (52). The substitutions chosen for study ranged from Y253A, which dramatically decreased TβRII binding, to I238A and Y252A which led to significant, but not as severe, reductions and I256A which led to no reduction. The substitutions were studied in the context of TGM constructs lacking domains 4 and 5 and were produced using mammalian cell expression, as done previously for full-length TGM and truncated forms, including TGM D1-D3 (15, 35). The results were overall consistent with those from the SPR binding studies, with the Y253A substitution blunting the signaling, except at the highest doses tested, and the I238A and Y252A substitutions, diminishing the signaling potency (EC_50_ 9.7 and 7.0 ng/ml, respectively, vs. 3.9 ng/ml for WT), but to a lesser degree than the Y253A substitution. The I256A substitution, which led to no reduction in TβRII binding affinity, was essentially equipotent with respect to WT (EC_50_ 4.5 ng/ml vs. 3.9 ng/ml for WT).

## Discussion

The genome of the mouse helminth *H. polygyrus* encodes a highly expanded family of CCP-containing proteins, several of which have been identified in its secretome to regulate host immune responses (15, 37, 39, 53). TGM, together with its five adult (TGM-2 to -6) and four larval (TGM-7 to -10) homologs, are among the proteins in this family, and at least two of these, TGM and TGM-2, have been shown to regulate immunosuppressive signaling through the T_reg_ pathway (35). Though potency of signaling through TGM is similar to that of TGF-β (15, 16), protein–protein binding kinetics and the amplitude and kinetics of signaling in murine reporter cell lines and primary murine T cells is distinct, with increased T_reg_ potency and decreased fibrotic gene response (15, 16).

The results presented here demonstrate that TGM binds the TGF-β receptors in a modular manner, with TGM-D2 and TGM-D3 as the main partners for TβRI and TβRII, respectively. The binding of TβRI is potentiated by TGM-D1, and this is likely mediated by a composite interface formed by both TGM-D1 and TGM-D2, not allostery, as the NMR titration data presented in Fig. S5*C* show that TGM-D1 directly, albeit weakly, binds TβRI. It is common for CCP-containing proteins to bind partners through arrays of CCPs, with avidity playing an important role (34). In addition, in multidomain CCP-containing proteins, the domains tend to be connected by short linkers and assume a relatively defined orientation to one another. In TGM, the linker connecting D1-D2 and D2-D3 is limited to just a few residues (Fig. S1). In addition, if one excludes the first four residues of TGM-D3, which are an artifact of the way the domain was produced, the entire domain, including the N- and C-termini, is overall quite rigid (Fig. 5*C*). The CCP domains that comprise TGM may therefore form a relatively extended structure with the domain orientations restricted to one another. In the case of TGM domains 1 and 2, this may be important for forming the shared interface that recognizes and binds TβRI. In the case of TGM domains 2 and 3, this may be important in positioning the type I and type II receptors with an appropriate spacing, and possibly also orientation, to enable efficient transphosphorylation and signaling.

The assembly of TβRI:TβRII signaling heterodimers by TGM is distinct compared to TGF-β homodimers, which assemble a (TβRI:TβRII)_2_ heterotetramer, first by binding TβRII with moderate to high affinity (K_D_ ca. 50 nM) and in turn by recruiting and binding TβRI through a composite TGF-β:TβRII interface (K_D_ ca. 30 nM) (18, 54, 55, 56). Though further studies are required, differences in kinetics of assembly, as well as the stoichiometry of the TGM vs. TGF-β signaling complexes, might account for at least some of the differences in the amplitude and kinetics of signaling that have been observed. These differences might also contribute to TGM’s gene expression profile, which is skewed away from extracellular matrix accumulation toward immunosuppression. Though domains 4 and 5 of TGM do not appear to be involved in ligating the TGF-β receptors, they might have other roles, such as targeting TGM to T cells or other cell types to enable effective immunosuppression *in vivo*.

The ITC competition experiments and NMR assignments of the free and bound forms of TβRI and TβRII demonstrate that TGM-D2 and TGM-D3 mimic TGF-β by engaging the same primary motifs of the receptors: the -PRDRP- prehelix extension, β5, and the extended C-terminus in TβRI and the β4 edge strand, as well as flanking acidic residues, Asp^55^ and Glu^142^, in TβRII. The fact that TGM-D2 engages not only the same regions of TβRI as both TGF-β and TβRII, but also an additional region, namely the C-terminal end of β1 and the turn that follows, suggests that this domain alone has extensively adapted to enable TGM’s high affinity for TβRI. This affinity is notably further augmented by TGM-D1, which evidently must recognize and bind TβRI at sites other than those bound by TGM-D2.

TGM-D3 is distinct from almost all other reported CCP domains in that its HVL is significantly extended (15). The structure of TGM-D3 and ^15^N T_2_ measurements show that the HVL extends laterally around the domain and is structurally ordered. The N-terminal end of the HVL packs against a triad of aromatic residues that protrude from the non-NC-face of the protein, including Tyr^192^, His^218^, and Phe^235^. This may serve to rigidify this portion of the HVL and position it to engage TβRII. The structure of TGM-D3 further shows that it is expanded laterally compared to canonical CCP domains. This is due to the elimination of the β′ and β’’ strands, which allows the C-terminal end of β4 and the extended segment that follows to diverge away from β1 and the extended N-terminus, against which it packs in canonical CCP domains. The expansion of the domain leads to partial exposure of several hydrophobic residues on both the NC- and non-NC-faces. These structural modifications of TGM-D3, together with the tentative identification of the binding site for TβRII on the non-NC-face of the protein created by these modifications, suggest that TGM-D3 accommodates TβRII by engaging its edge β-strand, β4, through hydrophobic residues on non-NC-face and that it stabilizes TβRII by interacting with Asp^55^ and Glu^142^ that flank β4. The basic residues on TGM-D3 that interact with Asp^55^ and Glu^142^ were potentially identified, as Arg^198^ on the N-terminal end of the HVL and Lys^254^ on the C-terminal tail. If proven by direct structural analysis, this would provide a remarkable demonstration of how TGM-D3 has adapted, relative to canonical CCP domains, to uniquely and specifically bind TβRII in a way that closely mimics that of the mammalian cytokine.

Though all domains of TGM are predicted to have the overall CCP fold, only TGM-D3 binds to TβRII. Sequence comparisons of TGM-D3 with the other domains of TGM show that they all contain two disulfide bonds and the HVL insertion (Fig. S12*A*). TGM-D3 is however unique in that the β3-β4 loop is 5 to 6 residues longer than other domains, suggesting that this loop is likely a tight β-turn in the other domains rather than a more extended turn, as in TGM-D3 (Fig. 5, *A* and *B*). This may alter the overall shape and dimensions of the C-terminal half of the protein to accommodate other binding partners. Most of the TGM-D3 residues that contribute 4-fold or greater to TβRII binding affinity, Phe^235^, Val^236^, Ile^238^, Tyr^252^, and Tyr^253^, are also divergent in the other domains, except for domain 1, and thus, these differences likely contribute to specific binding of TβRII by domain 3. However, Arg198 and Lys254 that may interact with TβRII Asn^55^ and Glu^142^ are divergent in domain 1 and thus may also impart D3 with specific binding to TβRII.

Though the TGM family is not fully characterized, TGM-2 and TGM-3 have been shown to also possess activity in TGF-β reporter gene assay in mouse fibroblasts and TGM-2 has been shown to possess T_reg_ conversion activity (35). Domain 3 sequence alignments show that all TGM homologs share overall high conservation, particularly among the four β-strands, the loop connecting β2 and β3, and the extended HVL. Residues shown to contribute more than 4-fold to binding, Arg^198^, Phe^235^, Val^236^, Ile^238^, Tyr^252^, Tyr^253^, and Lys^254^ are also fully conserved, with the only exceptions being Phe^235^ which is substituted with leucine in TGM-7 and Lys^254^ which is substituted with serine in TGM-4 and TGM-5, proline in TGM-6, and histidine in TGM-7. Hence, it is possible that domain 3 of all TGM homologs bind TβRII, though further studies are required to determine if this is correct, and if so, how the relative affinities compare.

The structural modifications demonstrated for TGM-D3 might extend to other CCP-containing proteins in HES. *Hp*ARI, and *Hp*BARI, for example, have three and two CCP domains, respectively (37, 38, 39) and except for domain 1 of *Hp*ARI and *Hp*BARI, which are 63 and 64 amino acids, respectively, all are of similar length to TGM-D3 (TGM-D3, HpARI CCP2, HpARI CCP3, and HpBARI CCP2 are 86, 86, 86, and 81 residues, respectively). HpARI CCP2 and CCP3 have been shown to be responsible for binding IL-33, while CCP1 of HpARI has been shown to bind DNA (38). Hence, the protein-binding domains of HpARI appear to be of similar length to TGM-D3 and thus these might also possess modifications, relative to canonical CCP domains, that impart them with their ability to bind IL-33. It is also possible that this is so for HpBARI, though this awaits direct demonstration that domain 2 is responsible for binding the IL-33 receptor, ST2.

TGM and domain-deleted forms thereof may have therapeutic potential for treating autoimmune disorders and as TGF-β signaling antagonists, respectively. The potential of TGM for treating autoimmune disorders has already been demonstrated in an animal model of colitis (57), though further work in this area is required to determine if TGM is equally as effective in expanding suppressive T_regs_ in humans as in mice and to develop strategies to mitigate formation of neutralizing antibodies. TGMs that include D1-D2 or D3, and lack either D3 or D1-D2, respectively, may be used to sequester TβRI or TβRII, thus functioning as competitive receptor antagonists to block signaling. These antagonists have significant potential for attenuating both soft-tissue cancers and the tissue fibrosis that are driven by dysregulated TGF-β signaling, though as with other TGF-β antagonists, strategies to reduce adverse consequences of on-target inhibition in vital tissues such as the heart must be considered (58, 59).

There are a number of human helminth parasites, including the nematodes *Necator americanus* and *Strongyloides stercoralis*, and the flatworms *Schistosoma mansoni* and *Taenia solium*, which achieve host immunomodulation by upregulating T_regs_. Though genome sequences are available, bioinformatic analyses have failed to identify any analogs of TGM, or related CCP-containing immunomodulatory proteins, such as Hp-ARI and Hp-BARI. This suggests that the expansion of the CCP-containing family in *H. polygyrus* is unique to this parasite and that the other parasites noted earlier upregulate T_regs_ by other mechanisms. Some true TGF-β family homologs, including TGH-2 from the human parasite *Brugia malayi* and FhTLM from the parasite *Fasciola hepatica*, have been implicated in the TGF-β signaling pathway, but these proteins have not yet been thoroughly characterized, either functionally or structurally (3, 4, 5).

The findings presented highlight the unique nature of *H. polygyrus*-mediated immunomodulation through the CCP domain–containing protein TGM. They show that although TGM is structurally dissimilar to TGF-β, it nonetheless engages the same binding sites on the type I and type II receptors as mammalian TGF-β, thereby mimicking the mammalian cytokine not only functionally but also molecularly. While structural studies of CCPs have demonstrated remarkable versatility in binding partners, none of the CCP domain structures reported to date have the dramatic structural modifications found in TGM-D3. Though further studies are required, these adaptations may be restricted to not only TGM but also other immunomodulatory CCP-containing proteins in the *H. polygyrus* secretome. These adaptations might have arisen owing to the strong selective pressure that must exist to allow a parasite to coexist within its host.

## Experimental procedures

### Expression and purification of TGM domains

DNA fragments corresponding to individual domains of *H. polygyrus* TGM, TGM-D1, TGM-D2, TGM-D3, and TGM-D1D2 were inserted between KpnI and HindIII sites in the modified form of pET32a (EMD-Millipore) that included a KpnI site immediately following the coding sequence for the thrombin recognition sequence. The resulting constructs, which included a thioredoxin-hexahistidine tag-thrombin cleavage site-TGM domain coding cassette (Table S1), were overexpressed in BL21(DE3) cells (EMD-Millipore) cultured at 37 °C. Unlabeled samples for binding studies were produced on rich medium (LB), while ^15^N and ^15^N,^13^C samples for NMR studies were produced using minimal medium (M9) containing 0.1% ^15^NH_4_Cl (Cambridge Isotope Laboratories) or 0.1% ^15^NH_4_Cl and 0.3% U-^13^C-D-glucose (Cambridge Isotope Laboratories). Carbenicillin was included in the growth medium at 50 μg mL^−1^ to select for cells bearing the expression plasmid. Protein expression was induced by adding 0.8 mM IPTG when the light scattering at 600 nm reached 0.75.

Cell pellets from 3 L of culture were resuspended in 100 ml of lysis buffer (50 mM Na_2_HPO_4_, 100 mM NaCl, 5 mM imidazole, 10 μM leupeptin, 10 μM pepstatin, 1 mM benzamide, pH 8.0) and sonicated. Following centrifugation (20 min, 15000*g*), the pellet was washed with 50 ml of water, resuspended in 50 mM Na_2_HPO_4_, 100 mM NaCl, 5 mM imidazole, 10 μM leupeptin, 10 μM pepstatin, 1 mM benzamide, and 8 M urea, pH 8.0, and stirred overnight at 25 °C. The remaining insoluble material was removed by centrifugation, and the supernatant was loaded onto a 50-ml metal affinity column (Ni^++^-loaded chelating sepharose, GE Lifesciences) pre-equilibrated with 125 ml of resuspension buffer. The column was washed with 100 ml of resuspension buffer, and the bound protein was eluted by applying a linear gradient of resuspension buffer containing 0.5 M imidazole.

Protein from the eluted peak was treated with reduced glutathione (GSH) at concentration equal to 2 mM x V_F_/V_P_, where V_F_ is the final volume of the folding buffer and V_P_ is the volume of TGM protein to be added to the folding buffer. After a 30-min incubation at 25 °C, the protein was slowly diluted into folding buffer (0.1 M Tris, 1 mM EDTA, 0.5 mM oxidized glutathione [GSSG], pH 8.0) to a final concentration of 0.1 mg mL^-1^ and stirred for 12 to 16 h at 4° C. The folding mixture was concentrated using an Amicon stirred cell fitted with a 5000 MWCO ultracel filter (Millipore) and dialyzed into 25 mM Tris, pH 8.7, at 4 °C. Solid thrombin was added to a final concentration of 4 U per milligram of TGM domain and incubated overnight at 25 °C. Cleavage was stopped by the addition of 10 μM leupeptin, 10 μM pepstatin, and 100 μM PMSF, and after readjusting the pH to 8.7, the cleavage mixture was passed over a Ni^++^ chelating sepharose column equilibrated with water. Column flow-through and a subsequent water wash, which contained primarily the TGM domain, were collected. For the TGM-D1 and TGM-D1D2 domains, the flow-through was bound to a Source Q column (GE Lifesciences) equilibrated in 25 mM CHES, pH 9.0, and eluted with a 0 to 0.5 M NaCl gradient. For the TGM-D2 and TGM-D3 domains, the flow-through was adjusted to pH 5.0 by the addition of acetic acid, bound to a Source S column (GE Lifesciences) equilibrated in 5 mM sodium acetate, 2M Urea, pH 5.0, and eluted with a 0 to 0.5 M NaCl gradient. Masses of the TGM domains were measured by liquid chromatography electrospray ionization time-of-flight mass spectrometry (LC-ESI-TOF-MS, Bruker Micro TOF). TGM-FL was expressed in expi293 cells (Invitrogen) and purified by metal affinity chromatography as previously described (15).

### Expression and purification of TGF-β receptor and growth factor constructs

The TβRII and TβRI ectodomains, and the TGF-β3 homodimer, were expressed in *E. coli* at 37 °C in the form of insoluble inclusion bodies, refolded, and purified as previously described (60, 61, 62). The engineered TGF-β monomer, mmTGF-β27M, which retains high affinity binding to TβRII, but has significantly improved solubility relative to TGF-β1, TGF-β2, and TGF-β3 homodimers, was produced and purified using the same procedure previously described (43). Masses were verified by LC-ESI-TOF-MS.

### Expression and purification of biotinylated avi-tagged TβRI, T*β*RII, and TGM-D3

Avi-tagged TβRI, TβRII, and TGM-D3 were produced using constructs modified to include the amino acid sequence “GLNDIFEAQKIEWHE” at the C-terminus. Protein expression and purification was carried out using the same procedures described previously for the nontagged protein. Biotinylation was performed using BirA biotin ligase as previously described (63). Constructs were validated by LC-ESI-TOF-MS where addition of a single biotin increases the protein mass by 226.3 Da. Following biotinylation, the proteins were repurified using ion-exchange chromatography to remove the biotinylation reagents.

### Expression and purification TGM-1 D3, TGM-1 D13, and TβRII variants

Constructs coding for *H. polygyrus* TGM-D3 and TβRII described previously were modified to introduce the desired substitution using site-directed mutagenesis with Phusion polymerase (ThermoFisher) as previously described (64). The resulting clones were sequenced over the entirety of their coding sequences to confirm the substitution. Constructs coding mutated forms of TGM D1-D3 (TGM D13) were generated by synthesis of coding sequences for TGM D13, identical to those described previously for TGM-1 D13, but with the desired substitution and then inserted into AscI- and ApaI-digested pSec-Tag2 as described previously (35). Desired constructs, which code for TGM D13 downstream of a signal peptide and with a C-terminal myc-tag and hexahistidine tag, were transfected into suspension cultured expi293 cells, and after 5 days, the protein was purified from the conditioned medium by capturing it on a NiNTA column (Thermo, His-Pur). The purified TGM D13 was pooled, deglycosylated with PNGase-F, concentrated, and further purified on Superdex 200 16/60 column (GE Lifesciences).

### SPR measurements

SPR datasets with TGM domains binding to TβRI or TβRII were generated using a BIAcore X100 instrument (GE Lifesciences) with biotinylated avi-tagged TβRI or TβRII captured onto neutravidin-coated CM-5 sensor chips (GE Lifesciences) at a density of 50 to 150 RU. Neutravidin-coated sensor chips for capture of biotinylated avi-tag receptors were made by activating the surface of a CM-5 chip with EDC and NHS, followed by injection of neutravidin (Pierce) diluted into sodium acetate at pH 4.5 until the surface density reached 6000 to 15,000 RU. Kinetic binding assays were performed by injections of the analytes in 25 mM Hepes, pH 7.4, 150 mM NaCl, 0.005% surfactant P20 (Pierce) at 100 μl min^−1^. Regeneration of the surface was achieved by a 30-s injection of 1 to 4 M guanidine hydrochloride. Baseline correction was performed by subtracting the response from both the reference surface with no immobilized ligand and 5 to 10 blank buffer injections. Kinetic analyses were performed by fitting the results from a single injection series to a simple 1:1 model using the program Scrubber (Biologic Software).

SPR datasets with TGM-D3 and TβRII variants were generated in the same overall manner described previously, using either biotinylated avi-tagged TβRII or biotinylated avi-tagged TGM-D3 captured at a density of 50 to 150 RU onto neutravidin-coated CM-5 sensor chips (GE Lifesciences). Kinetic binding assays were performed by injections of the analytes in 25 mM Hepes, pH 7.4, 150 mM NaCl, 0.05% surfactant P20 (Pierce) at 100 μl min^−1^. Regeneration of the surface was achieved by a 30-s injection of 100 mM – 200 mM guanidine hydrochloride. Baseline correction was performed by subtracting the response from both the reference surface with no immobilized ligand and 5 to 10 blank buffer injections. Kinetic analyses were performed by fitting the results from duplicate or triplicate injection series to a simple 1:1 model using the program Scrubber (Biologic Software).

### ITC experiments

ITC datasets were generated using a Microcal PEAQ-ITC instrument (Malvern Instruments). All experiments with TβRII were performed in 25 mM sodium phosphate, 50 mM NaCl, pH 6.0, at a temperature of 35 °C, while all experiments with TβRI were performed in 25 mM Hepes, 50 mM NaCl, 0.05% NaN_3_, pH 7.5, at a temperature of 25 °C. Proteins included in the syringe and sample cell were dialyzed against ITC buffer and concentrated as necessary prior to being loaded into either the syringe or the sample cell. Protein concentrations in the cell and syringe are indicated in Table S2. TβRII experiments were carried out with 15 2.5-μl injections with an injection duration of 5 s, a spacing of 150 s, and a reference power of 10, while TβRI experiments were carried out with 19 2.0-μl injections with an injection duration of 4 s, a spacing of 150 s, and a reference power of 10. Integration and data fitting were performed using the programs Nitpic (65), Sedphat (66, 67), and GUSSI (68).

ITC competition experiments with TβRII were performed in 25 mM sodium phosphate, 50 mM NaCl, pH 6.0, at 35 °C, while those with TβRI were performed in 25 mM Hepes, 50 mM NaCl, 0.05% NaN_3_, pH 7.5, at 25 °C, with exception of the TβRI TGF-β(TβRII)_2_ titration which was performed at 30 °C. Protein concentrations in the cell and syringe are indicated in Tables S2 and S3. The TβRII mmTGF-β27M/TGM-D3 competition experiments were performed with 13 3.0-μl injections with an injection duration of 5 s, a spacing of 150 s, and a reference power of 10, as was the TβRI TGF-β(TβRII)_2_ titration. The TGM-D12 TGF-β(TβRII)_2_(TβRI)_2_ titration was performed with 19 2.0-μl injections with an injection duration of 4 s, a spacing of 150 s, and a reference power of 10. The integration and data fitting were performed as stated previously.

### NMR sample preparation and 1D/2D experiments

Samples of TGM-D1, TGM-D2, TGM-D3, and corresponding complexes with TβRI and TβRII, for NMR were prepared at a concentration of 0.03 to 0.2 mM in 25 mM Na_2_HPO_4_, 50 mM NaCl, pH 6.0, and transferred to 5-mm susceptibility-matched microtubes (Shigemi) for data collection. NMR data were collected at 30 °C using a Bruker 600, 700, or 800 MHz spectrometer equipped with a 5-mm ^1^H (^13^C,^15^ N) z-gradient “TCI” cryogenically cooled probe (Bruker Biospin). One-dimensional ^1^H spectra were acquired with an excitation sculpting water suppression scheme (69). Two-dimensional ^1^H-^15^ N HSQC spectra were acquired with a sequence with water flipback pulses (70) and WATERGATE water suppression pulses (71). To probe conformational exchange, ZZ-exchange experiments were recorded with ^15^N TGM-D2 as previously described (72). NMR data were processed using nmrPipe (73) and analyzed using NMRFAM-SPARKY (74).

### NMR ^15^N T_2_ calculations

^15^N T_2_ backbone amide relaxation times for TGM-D3 were measured at 310 K using the interleaved pulse sequence as described previously (75). The ^15^N T_2_ data were collected using 8 delay times ranging from 16 to 240 msec. To calculate the ^15^N T_2_ relaxation time per residue, a two-parameter decaying exponential was used to fit the relative peak intensities as a function of delay time. The sample was prepared in 25 mM MES, 50 mM NaCl, pH 6.0.

### NMR backbone assignment

Backbone resonances were assigned by recording and analyzing 2-D ^1^H-^15^N HSQC and 3-D HNCACB, CBCA(CO)NH, HNCA, HN(CO)CA, HNCO, and HN(CA)CO triple resonance datasets. Proton and side chain resonances were assigned by recording and analyzing 2-D ^1^H-^13^ C CT-HSQC and 3-D CC(CO)NH, HBHACONH, HCCH-TOCSY, H(CC, CO)NH, HNHA, and HNHB datasets. NMR data were processed using nmrPipe (73) and analyzed using a combination of NMRFAM-SPARKY (74), PINE (76, 77), and PECAN (49).

### NMR chemical shift perturbation calculations

Backbone resonances were assigned for TβRI and TβRII, both free and bound to TGM-D2 and TGM-D3, respectively. The absolute value of the chemical shift differences was calculated for each of the backbone nuclei (^15^N^H^, ^1^H^N^, ^1^H^α^, ^13^C^α^, ^13^C^β^, ^13^C^O^) and then normalized to the largest shift perturbation. These values were summed for each residue and then normalized according to the number of nuclei that contributed to the final shift perturbation value.

### NMR structure determination of TGM-D3

The solution structure of TGM-D3 was calculated using the program NIH-XPLOR (78) with assigned ^1^H-^1^H NOEs, ^1^H-^15^N, ^1^H^α^-^13^C^α^ and ^13^C^α^−^13^C^O^ residual dipolar couplings (RDCs), TALOS-derived phi and psi restraints (79), hydrogen bond restraints, and ^3^J^HN-Hα^ J-coupling restraints as input. The ^1^H-^1^H distance restraints were derived from manually peak-picked 3D ^15^N-edited and 3D ^13^C-edited NOESY datasets using the program CCP-NMR (80), with distance restraints derived using routines provided by CCP-NMR. The RDCs were recorded using a sample with 10 mg mL^-1^ Pf1 phage for alignment (81) and were measured using a 2-D IPAP-HSQC (46) for ^1^H-^15^N RDCs, a 3D ^13^C^α^-coupled HNCO for ^13^C^α^−^13^C^O^ RDCs, and a 3D H^α^-coupled HN(CO)CA for ^1^Hα-^13^Cα RDCs. The ^3^J^HN-Hα^ was measured from the ratio of the crosspeak to diagonal in a 3D H^N^-H^α^ experiment as described (82). Ramachandran analysis was performed using the program PROCHECK (83, 84).

### TGF-β reporter bioassay

The TGF-β bioassay (cell line clone MFB-F11) developed by Tesseur *et al*. (52) was performed as previously described (15). Confluent cells were detached with trypsin and resuspended in DMEM with 2.5% fetal calf serum, 100 U/ml penicillin, 100 μg/ml streptomycin, and 2 mM L-glutamine at a concentration of 8 × 10^5^ cells/ml. In 50 μl, 4 × 10^4^ cells were added to each well of a 96-well flat-bottomed plate. Dilutions of purified proteins were then added to each well in a volume of up to 50 μl and incubated for 24 h at 37 °C. Subsequently, 20 μl of the supernatant was aspirated from each well, added to an ELISA plate (Nalge Nunc International) with 180 μl of reconstituted Sigma FastTM p-nitrophenyl phosphate substrate, and incubated at room temperature (RT) in dark for up to 18 h. Plates were read on at 405 nm on an E_max_ precision microplate reader (Molecular Devices). All conditions were set up in triplicate.

## Data availability

The assigned chemical shifts for the TGM-D2 bound form of TβRI, the TGM-D3 bound form of TβRII, TβRII-bound TGM-D3, and unbound TGM-D3 have been deposited to BioMagResBank under accession codes 51083, 51084, 51085, and 51086, respectively. The structures, and accompanying restraints, of TGM-D3 have been deposited to the RCSB PDB under accession code 7SXB.

## Supporting information

This article contains supporting information.

## Conflicts of interest

The authors declare that they have no conflicts of interest with the contents of this article.
