# Supplementary material for: Convergent evolution of a parasite-encoded complement control protein-scaffold to mimic binding of mammalian TGF-β to its receptors, TβRI and TβRII
Source: J Biol Chem. 2022 Apr 29;298(6):101994. doi: 10.1016/j.jbc.2022.101994 (PMC9163516; doi:10.1016/j.jbc.2022.101994)

Supporting Information

Convergent evolution of a parasite-encoded complement control protein-scaffold to mimic binding of mammalian TGF-β to its receptors, TβRI and TβRII

Ananya Mukundan, Chang-Hyeock Byeon, Cynthia S. Hinck, Kyle Cunningham, Tiffany Campion, Danielle J. Smyth, Rick M. Maizels, and Andrew P. Hinck

Materials included: 5 Tables and 12 Figures

Table S1. *H. polygyrus* constructs used in this study

Construct	Coding region and description (* indicates stop codon)
TGM-D1	Residues 16-95 of *H. polygyrus* TGF-β Mimic, NCBI MG099712<br><br>Thioredoxin-His_6-Linker-Thrombin Cleavage Site-Linker-TGM-D1<br><br>MSDKIIHLTDDSFDTDVLKADGAILVDFWAEWCGPCKMIAPILDEIADEYQGKLTVAK<br>LNIDQNPGTAPKYGIRGIPTLLLFKNGEVAATKVGALSKGQLKEFLDANLAGSGSGHM<br>HHHHHHSSGLVPRGSGTGSGSGSDDSGCMPFSDEAATYKYVAKGPKNIEIPAQIDNSG<br>MYPDYTHVKRFCKGLHGEDTTGWFVGICLASQWYYYEGVQECDDR*
TGM-D2	Residues 96-176 of *H. polygyrus* TGF-β Mimic, NCBI MG099712<br><br>Thioredoxin-His_6-Linker-Thrombin Cleavage Site-Linker-TGM-D2<br><br>MSDKIIHLTDDSFDTDVLKADGAILVDFWAEWCGPCKMIAPILDEIADEYQGKLTVAK<br>LNIDQNPGTAPKYGIRGIPTLLLFKNGEVAATKVGALSKGQLKEFLDANLAGSGSGHM<br>HHHHHHSSGLVPRGSGTRCSPLPTNDTVSFEYLKATVNPGIIFNITVHPDASGKYELTYI<br>KRICKNFPTDSNVQGHIIGMCYNAEWQFSSTPTCPAS*
TGM-D12	Residues 16-176 of *H. Polygyrus* TGF-β Mimic, NCBI MG099712<br><br>Thioredoxin-His_6-Linker-Thrombin Cleavage Site-Linker-TGM-D12<br><br>MSDKIIHLTDDSFDTDVLKADGAILVDFWAEWCGPCKMIAPILDEIADEYQGKLTVAK<br>LNIDQNPGTAPKYGIRGIPTLLLFKNGEVAATKVGALSKGQLKEFLDANLAGSGSGHM<br>HHHHHHSSGLVPRGSGTGSGSGSDDSGCMPFSEAATYKYVAKGPKNIEIPAQIDNSGM<br>YPDYTHVKRFCKGLHGEDTTGWFVGICLASQWYYYEGVQECDDRRCSPLPTNDTVSF<br>EYLKATVNPGIIFNITVHPDASGKYPELTYIKRICKNFP<br>TDSNVQGHIIGMCYNAEWQFSSTPTCPAS*
TGM-D3	Residues 177-262 of *H. Polygyrus* TGF-β Mimic, NCBI MG099712<br><br>Thioredoxin-His_6-Linker-Thrombin Cleavage Site-Linker-TGM-D3<br><br>MSDKIIHLTDDSFDTDVLKADGAILVDFWAEWCGPCKMIAPILDEIADEYQGKLTVAK<br>LNIDQNPGTAPKYGIRGIPTLLLFKNGEVAATKVGALSKGQLKEFLDANLAGSGSGHM<br>HHHHHHSSGLVPRGSGTGCPPLPDDGIVFYEYYGYAGDRHTVGPVVTKDSSGNYPSPT<br>HARRRCRALSQEADPGEFVAICYKSGTTGESHWEYYKNIGKCPDP*
TGM-D13	Residues 16-262 of *H. Polygyrus* TGF-β Mimic, NCBI MG099712<br><br>Signal Peptide-TGM-D1-D3-Linker-Myc-Linker-His_6<br><br>METDTLLLWVLLLWVPGSTGDAAQPARRADDSGCMPFSDEAATYKYVAKGPKNIEIP<br>AQIDNSGMYPDYTHVKRFCKGLHGEDTTGWFVGICLASQWYYYEGVQECDDRRCSPL<br>PTNDTVSFEYLKATVNPGIIFNITVHPDASGKYPELTYIKRICKNFPTDSNVQGHIIGMCY<br>NAEWQFSSTPTCPASGCPPLPDDGIVFYEYYGYAGDRHTVGPVVTKDSSGNYPSPTHA<br>RRRCRALSQEADPGEFVAICYKSGTTGESHWEYYKNIGKCPDPGPEQKLISEEDLNSAV<br>DHHHHHH*
TGM-FL	Residues 16-422 of *H. Polygyrus* TGF-β Mimic, NCBI MG099712<br><br>Signal Peptide-TGM-FL-Linker-Myc-Linker-His_6<br><br>METDTLLLWVLLLWVPGSTGDAAQPARRADDSGCMPFSDEAATYKYVAKGPKNIEIP<br>AQIDNSGMYPDYTHVKRFCKGLHGEDTTGWFVGICLASQWYYYEGVQECDDRRCSPL<br>PTNDTVSFEYLKATVNPGIIFNITVHPDASGKYPELTYIKRICKNFPTDSNVQGHIIGMCY<br>NAEWQFSSTPTCPASGCPPLPDDGIVFYEYYGYAGDRHTVGPVVTKDSSGNYPSPTHA<br>RRRCRALSQEADPGEFVAICYKSGTTGESHWEYYKNIGKCPDPRCKPLEANESVHYEY<br>FTMTNETDKKKGPPAKVGKSGKYPEHTCVKKVCSKWPYTCSTGGPIFGECIGATWNFT<br>ALMECINARGCSSDDLFDKLGFEKVIVRKGEGSDSYKDDFARFYATGSKVIAECGGKT<br>VRLECSNGEWHEPGTKTVHRCTKDGIRTLGPEQKLISEEDLNSAVDHHHHHH*

Table S2. TGM:TβRI and TGM:TβRII binding as assessed by ITC

Cell	TβRI	TβRI	TβRI	TβRI	TβRI	TβRII	TβRII	TβRII	TβRII
Syringe	TGM-D1	TGM-D2	TGM-D3	TGM-D1D2	TGM-FL	TGM-D1	TGM-D2	TGM-D3	TGM-FL
Cell concentration (μM)	7.5	7.5	7.5	7.5	7.5	15	15	15	15
Syringe concentration (μM)	150	150	135	100	58	300	300	300	320
Temperature (°C)	25	25	25	25	25	35	35	35	35
K_D (nM)	ND[a]	1500 (500 – 4600)[bc]	ND[a]	25 (11, 48)[bc]	52 (29 – 90)[bc]	ND[a]	ND[a]	1200 (900, 1500)[bd]	550 (260, 1080)[be]
ΔH (kcal mol^{-1})	ND[a]	-18 (-27– -13)[b]	ND[a]	-19 (-20 – -18)[b]	-17 (-18 – -15)[b]	ND[a]	ND[a]	-11 (-11, -10)[b]	-7.1 (-7.9, -6.6)[b]
ΔG (kcal mol^{-1})	ND[a]	-8.0	ND[a]	-11	-9.9	ND[a]	ND[a]	-8.4	-8.8
-TΔS (kcal mol^{-1})	ND[a]	9.7	ND[a]	8.3	6.8	ND[a]	ND[a]	2.4	-1.7
Stoichiometry (n)	ND[a]	0.54[f]	ND[a]	1.2[f]	0.96[f]	ND[a]	ND[a]	1.1[f]	0.84[f]

[a]Not determined due to weak signal

[b]Uncertainty reported as 68.3% confidence interval

[c]Fit for one replicate

[d]Global fit of three replicates

[e]Global fit of two replicates

[f]Number of sites determined by incompetent fraction value on sedphat; set to ‘1’ for K_D analysis

Table S3. ITC-based TβRI and TβRII competition binding

Cell	TβRI	TβRI	TβRII
Syringe	TGF-β(TβRII)$_2$	TGM-D1D2	mmTGF-β27M
Competitor[a]	None	6 μM TGF-β(TβRII)$_2$	0, 6.0, or 12.0 μM TGM-D3
Cell concentration (μM)	5	10	15
Syringe concentration (μM)	100	110	150
Temperature (°C)	30	25	35
K_D (nM)	61 (36 - 97)[d]	ND[b]	35 (17 - 64)[c, d]
ΔH (kcal mol^{-1})	-4.2 (-4.5 - -4.0)[d]	ND[b]	-7.4 (-7.7 - -7.0)[c, d]
ΔG (kcal mol^{-1})	-10	ND[b]	-11[e]
-TΔS (kcal mol^{-1})	-5.8	ND[b]	-3.2[e]

[a]Competitor was added to the sample cell

[b]K_D, ΔH, ΔG and -TΔS were unable to be fitted

[c]K_D and ΔH correspond to the parameters, derived from the global fit, for TβRII:mmTGF-β27M binding in the absence of competitor, uncertainty determined by 68.3% confidence interval

[d]Fit for one replicate

[e]ΔG and -TΔS correspond to those for TβRII:mmTGF-β27M binding in the absence of competitor calculated from ΔG = ΔH- TΔS and globally fitted values for K_D and ΔH

Table S4. TGM-D3 Structural Statistics	
**NOE**	
Intramolecular NOE: i-j = 0	465
Sequential NOE: i-j = 1	323
Short-Range NOE:1 < i-j <5	104
Long-Range: i-j ≥ 5	247
**Angle**	
TALOS (ϕ,φ) dihedral constraints	120
$^3J_{HNHA}\alpha$	39
**RDC**	12
RDC: N-H	69
RDC: Hα-Cα	74
RDC: Cα-CO	66
**RMSD (Deviations)**	
Bonds (Å)	0.008 ± 0.000
Impropers (°)	1.067 ± 0.159
Angles (°)	1.032 ± 0.035
Dihedral (°)	4.171 ±0.441
HBDA (Å)	0.025 ± 0.009
$^3J_{HNHA}\alpha$ (Hz)	1.349 ± 0.092
**Ramachandran**[a]	
Most Favored	81.2%
Additionally Allowed	11.6%
Generously Allowed	5.8%
Disallowed	1.4%
**RMSD**[b]	
Secondary Structure[c]	
Backbone	0.68Å
Heavy	1.14Å
Core[d]	
Backbone	1.00Å
Heavy	1.48Å

[a]Ramachandran values from the ten lowest-energy structures
[b]RMSD values are computed from a mean structure
[c]Residues 17-21, 45-49, 56-58, 62-69, 76-80
[d]Residues 6-81

Table S5. WT TGM-D3:TβRII variant and WT TβRII:TGM-D3 variant binding as assessed by SPR

Surface	Analyte	Fitted Parameters[a]			
		k_{on} (M^{-1} s^{-1})	k_{off} (s^{-1})	K_d (μM)	R_{max} (RU)
TGM-D3	WT TβRII	(4.1 ± 0.1) x 10^5	0.7 ± 0.1	1.6 ± 0.1	240 ± 10
TGM-D3	D55N	(5.0 ± 0.2) x 10^4	3.1 ± 0.1	63 ± 1	200 ± 10
TGM-D3	I73A	(1.6 ± 0.1) x 10^5	1.1 ± 0.1	6.9 ± 0.1	220 ± 10
TGM-D3	S75L	(1.3 ± 0.1) x 10^4	3.9 ± 0.9	310 ± 30	250 ± 20
TGM-D3	I76A	(4.7 ± 0.1) x 10^4	1.2 ± 0.1	26 ± 1	430 ± 10
TGM-D3	E142Q	(4.1 ± 0.1) x 10^5	10 ± 10	17± 1	130 ± 10
TβRII	WT TGM-D3	(1.6 ± 0.1) x 10^5	0.26 ± 0.01	1.6 ± 0.1	120 ± 10
TβRII	R198A	(1.1 ± 0.1) x 10^5	0.78 ± 0.01	70 ± 1	260 ± 10
TβRII	H199A	(3.3 ± 0.1) x 10^5	0.98 ± 0.01	3.0 ± 0.1	310 ± 10
TβRII	F235A	(4.5 ± 0.1) x 10^5	1.8 ± 0.2	4.1 ± 0.1	63 ± 1
TβRII	V236A	(3.8 ± 0.1) x 10^5	1.8 ± 0.1	4.6 ± 0.1	84 ± 1
TβRII	I238A	(9.4 ± 0.1) x 10^4	2.3 ± 0.1	25 ± 1	140 ± 10
TβRII	Y252A	(7.8 ± 0.2) x 10^4	1.7 ± 0.1	21 ± 1	150 ± 10
TβRII	Y253A	ND[b]	ND[b]	ND[b]	ND[b]
TβRII	K254A	(3 ± 2) x 10^5	12 ± 6	35 ± 1	310 ± 10
TβRII	N255A	(4.8 ± 0.1) x 10^5	1.3 ± 0.1	2.7 ± 0.1	150 ± 10
TβRII	I256A	(8.8 ± 0.1) x 10^5	1.4 ± 0.1	1.6 ± 0.1	130 ± 10
TβRII	K258A	(4.3 ± 0.1) x 10^5	1.2 ± 0.1	2.7 ± 0.1	250 ± 10

[a]Fitted parameters were derived from kinetic analysis of a duplicate or triplicate injection series
[b]Not determined due to weak signal

[a]Not determined due to weak signal

Figure S1: ITC thermograms for TGM binding to TβRI and TβRII. A-E. Raw thermograms for the injection of (A) TGM-D2, (B) TGM-D12, or (C) TGM-FL into TβRI, and (D) TGM-D3 or (E) TGM-FL into TβRII. **F-G**, **J-K**. Raw thermograms for the injection of (F) TGM-D1 or (G) TGM-D3 into TβRI, with corresponding integrated heats (J and K, respectively). **H-I, L-M.** Raw thermograms for the injection of (H) TGM-D1 or (I) TGM-D2 into TβRII, with corresponding integrated heats (L and M, respectively).

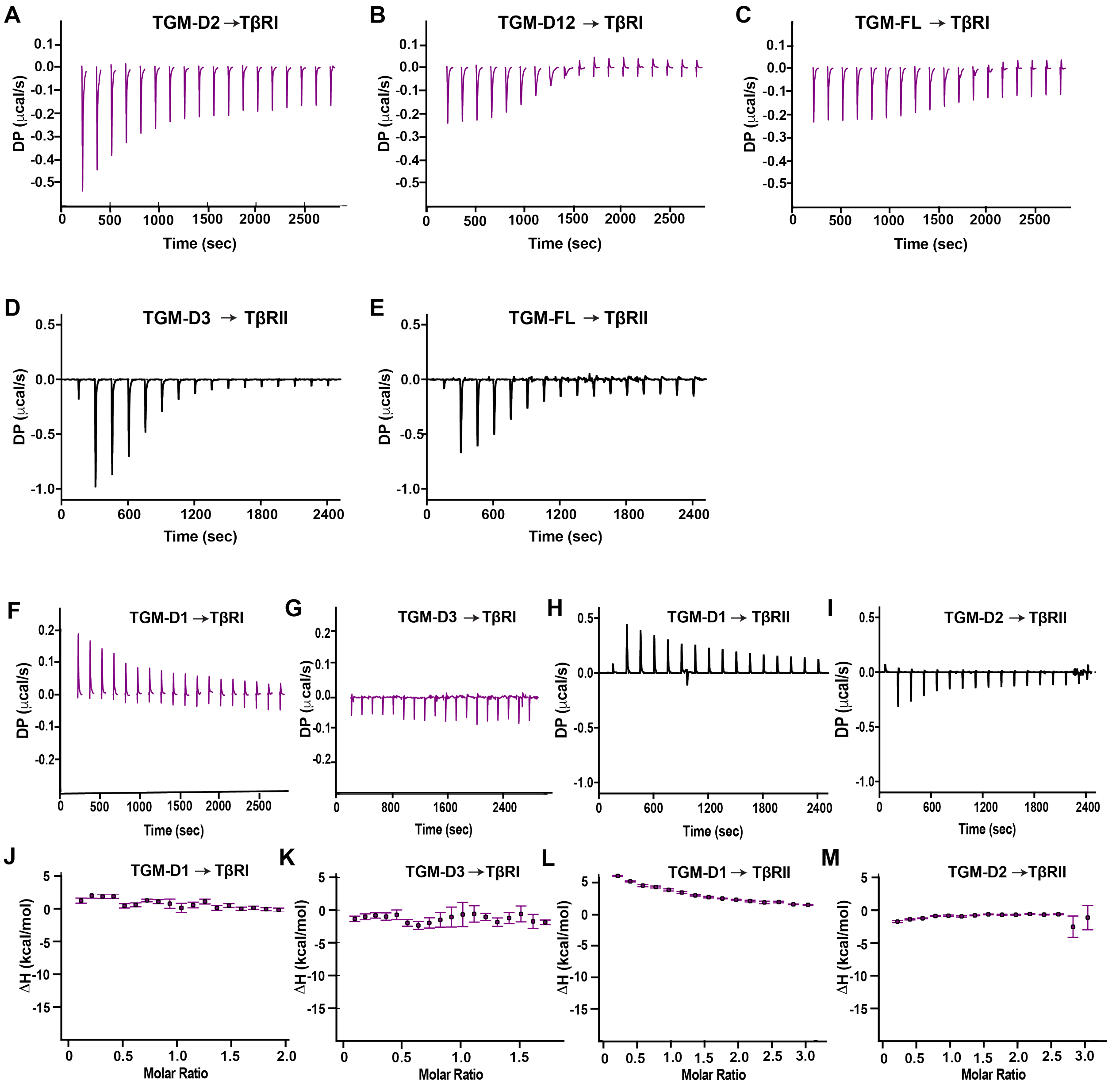

Figure S2. **^{1}H-^{15}N HSQC spectra of TGM-D2 and TGM-D3**. **A-B.** ^{1}H-^{15}N HSQC spectrum of ^{15}N TGM-D2 (A). Blue boxes mark doubled peaks in dynamic equilibrium with one another as identified by a ZZ-exchange HSQC experiment (expansion of ZZ-exchange HSQC spectrum with a mixing time of 250 ms is shown as an inset for two pairs of peaks). Peak expansion corresponding to ZZ-exchange HSQC experiment as a function of the mixing time is shown for the pair of peaks at ^{1}H 10.3 ppm/^{15}N 124 ppm (B). **C.** ^{1}H-^{15}N HSQC spectrum of ^{15}N TGM-D3. All spectra recorded in 25 mM sodium phosphate, 50 mM sodium chloride, 5% ^2H_2O pH 6.0, 310 K.

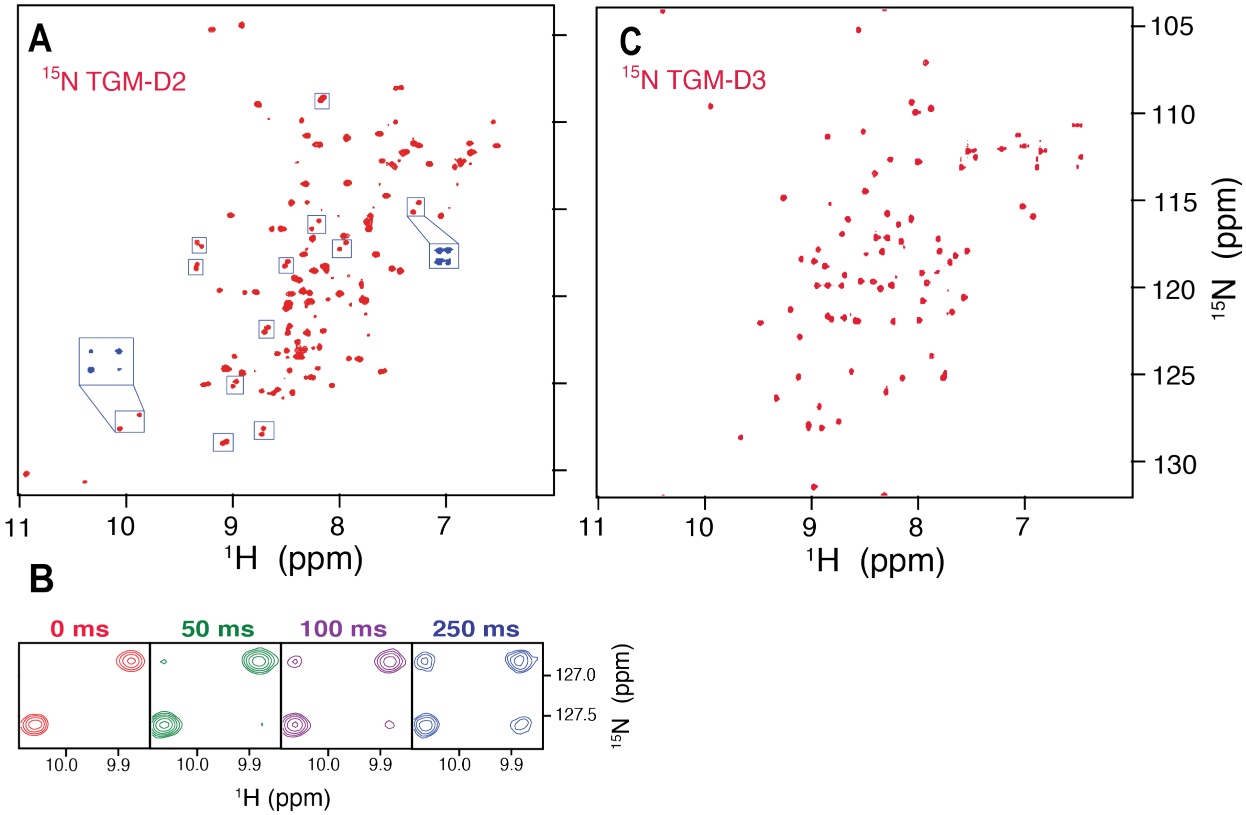

Figure S3. **^{1}H-^{15}N HSQC spectra of TGM-D1**. **A.** ^{1}H-^{15}N HSQC spectrum of 100 µM ^{15}N TGM-D1 in 25 mM sodium phosphate, 250 mM sodium chloride, 5% $^{2}H_2O$ pH 6.0, 310 K (A). **B-D.** ^{1}H-^{15}N HSQC spectrum of 200 µM ^{15}N TGM-D1 in the same buffer as panel A, but with a protein concentration of 200 µM and with 10 mM CHAPS added (B), a protein concentration of 20 µM ^{15}N TGM-D1 but no CHAPs (C), or a protein concentration of 20 µM and with 10 mM CHAPS added (D).

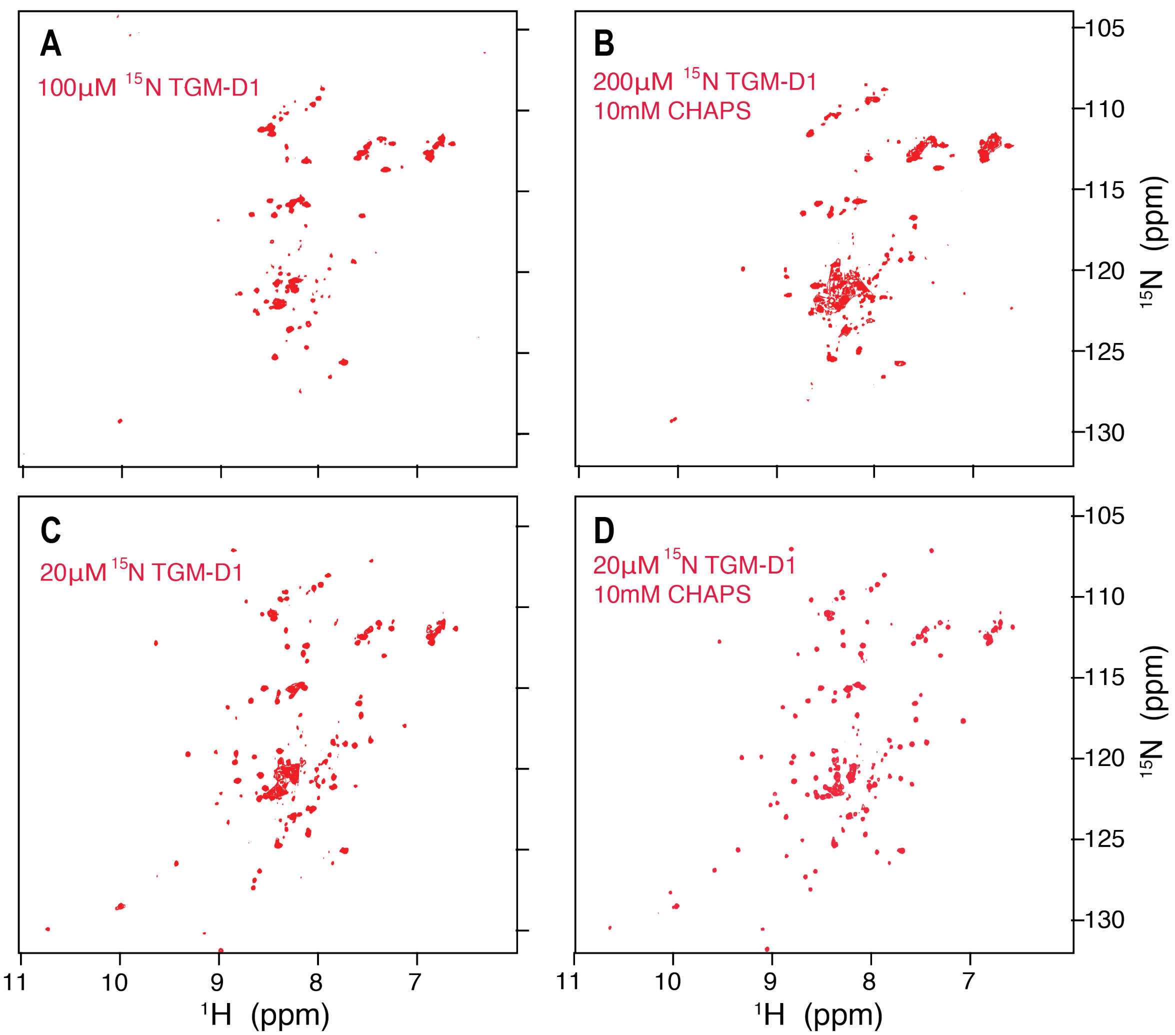

Figure S4. **Binding of TGM domains by TβRI. A-B.** ^{1}H-^{15}N HSQC spectra of TGM-D1 alone (red) or with 1.2 molar equivalents of unlabeled TβRI (blue) (A). ^{1}H-^{15}N HSQC spectra of TGM-D3 alone (red) or with 1.2 molar equivalents of unlabeled TβRI (blue) (B). **C-D.** ^{1}H-^{15}N HSQC spectra of TGM-D2 alone (C) or with 1.2 molar equivalents of unlabeled TβRI (D). The boxed regions on the spectra mark peaks in conformational exchange (C) or resolved into a single peak by TβRI binding (D). All spectra recorded in 25 mM sodium phosphate, 50 mM sodium chloride, 5% $^{2}H_2O$ pH 6.0, 310 K.

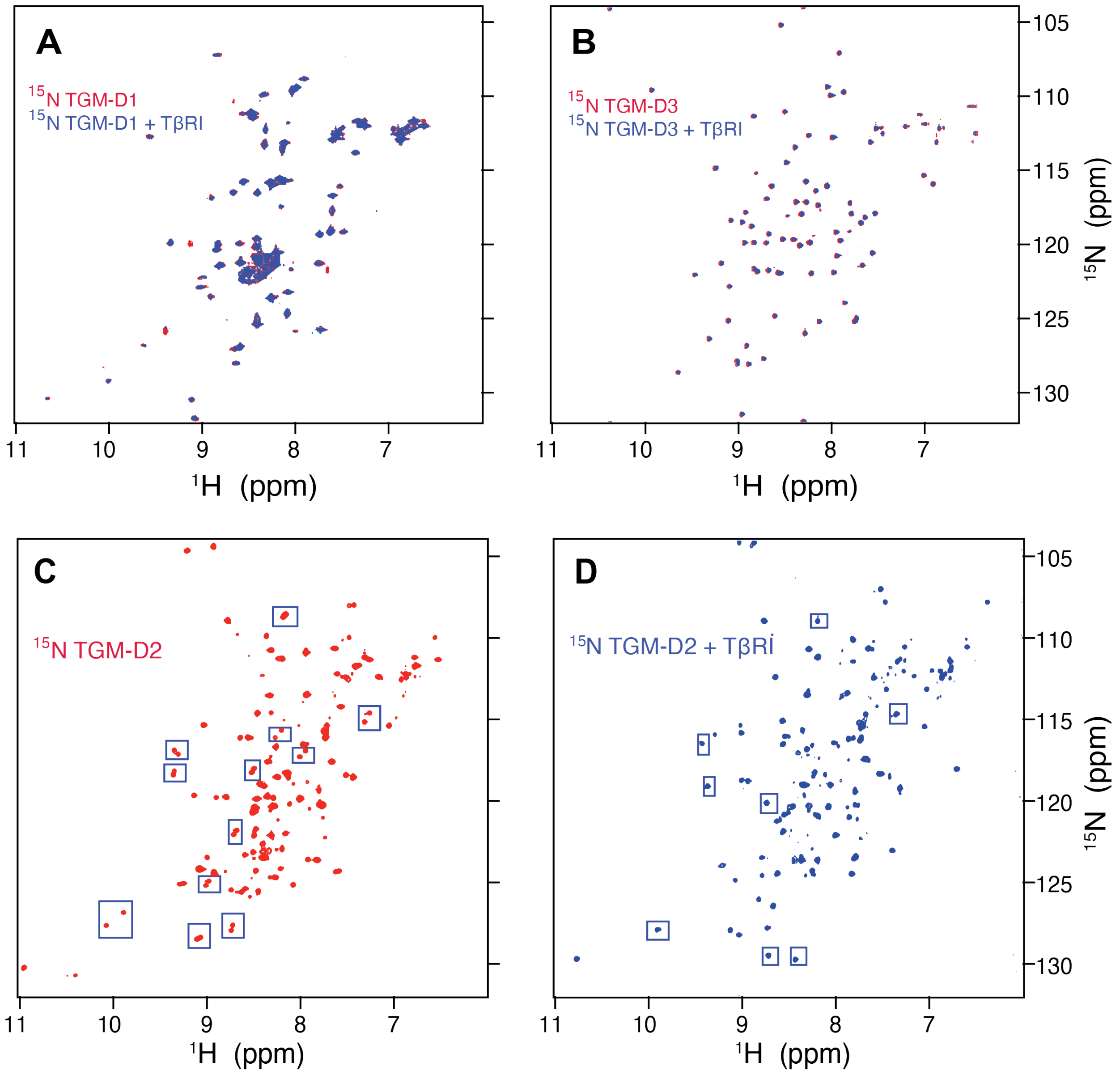

Figure S5. Binding of ^{15}N TβRI by TGM-D1, TGM-D2, and TGM-D3. A-B. ^{1}H-^{15}N HSQC spectra 0.03 mM ^{15}N TβRI alone (red) overlaid with the spectrum of the same sample but with 1.5 molar equivalents of unlabeled TGM-D2 (A) or TGM-D3 (B) added (blue). Expansion of boxed region in panel A at intermediate titration points is shown below panel A. **C.** ^{1}H-^{15}N HSQC spectrum of 0.03 mM ^{15}N TβRI alone (red) overlaid with the spectrum of the same sample but with 1.5 molar equivalents of unlabeled TGM-D1 added. The boxed inset at the top of panel C shows a plot of the intensity ratios ($I_{TGM\text{-}D1\text{-}bound}/I_{free}$) per residue of TβRI. The red dots on the baseline indicate residues that completely disappeared upon addition of TGM-D1 to ^{15}N TβRI. Boxed residues in the HSQC of panel C indicate residues of TβRI that undergo a chemical shift upon addition of titrating amounts of TGM-D1. Spectra recorded in 25 mM sodium phosphate, 50 mM sodium chloride, 5% $^{2}H_2O$ pH 6.0, 310 K.

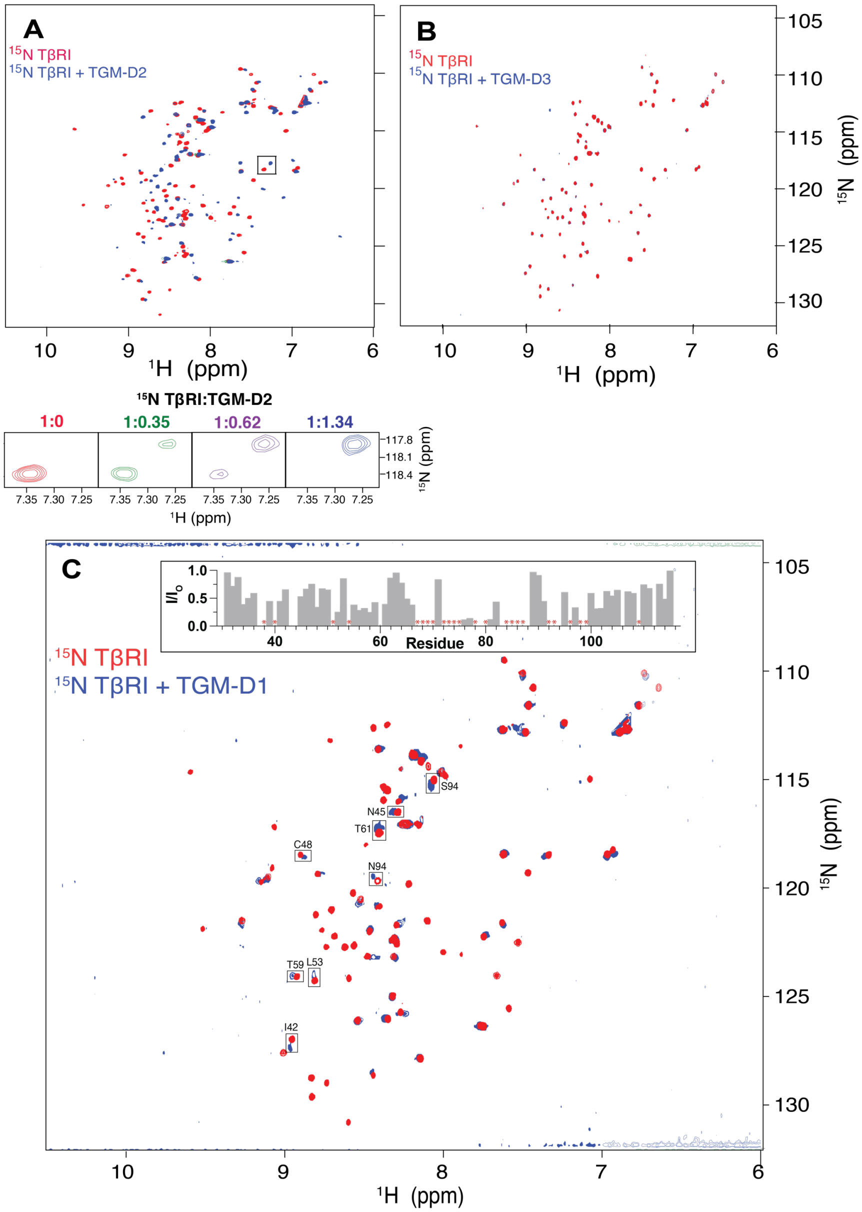

Figure S6. ^{1}H-^{15}N HSQC assignments of TβRI alone and bound to TGM-D2. A. ^{1}H-^{15}N HSQC spectra of TβRI alone with peaks assigned. **B.** ^{1}H-^{15}N HSQC spectra of TβRI bound to TGM-D2 with peaks assigned. Dashed horizontal lines in panel A indicate sidechain $-NH_2$ resonances of Asn/Gln residues. Spectra recorded in 25 mM HEPES, 50 mM sodium chloride, 0.02% azide, 5% $^{2}H_2O$ pH 6.0, 300K.

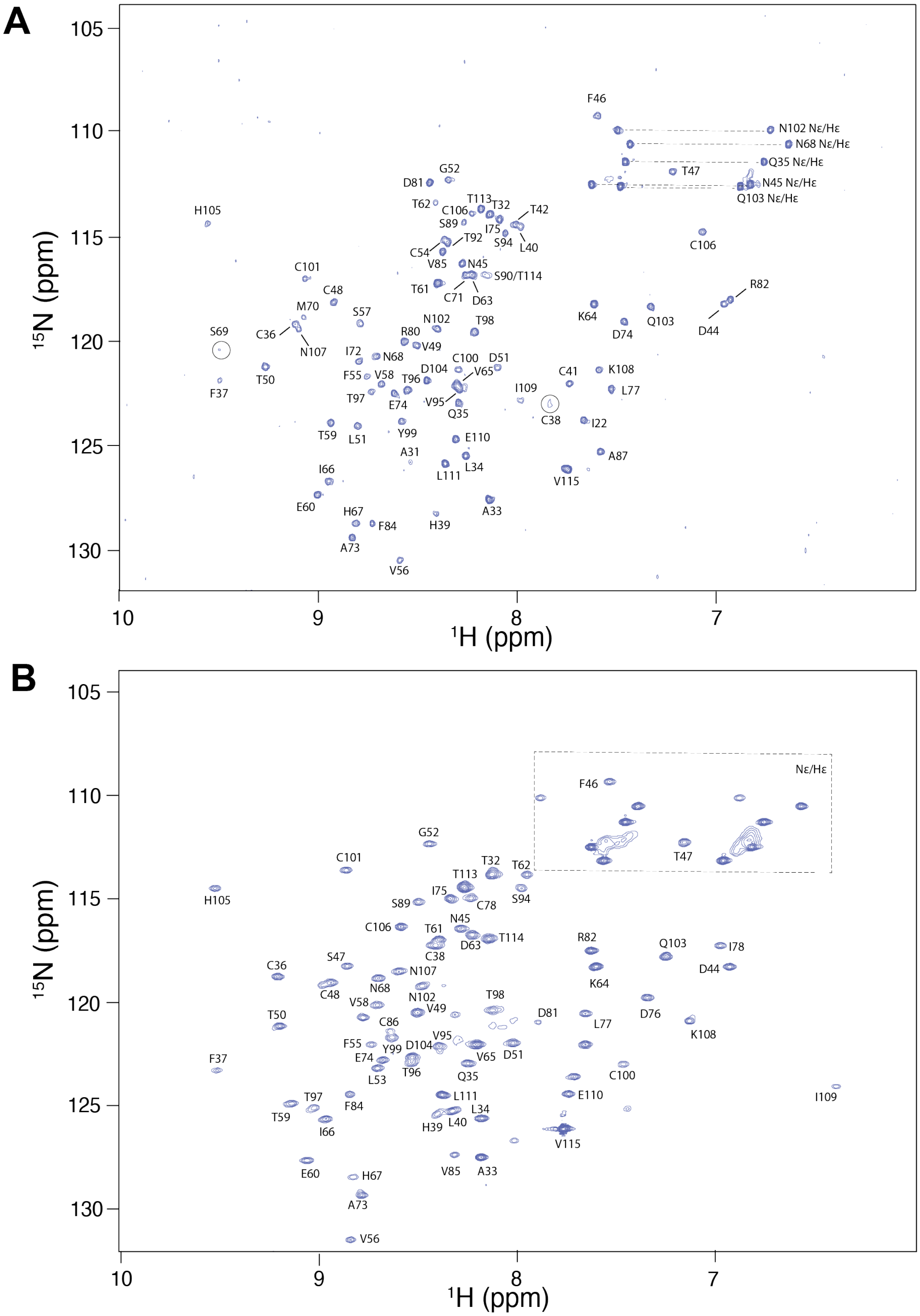

Figure S7. Binding of TGM-D1, TGM-D2, and TGM-D3 by TβRII. A-B. ^{1}H-^{15}N HSQC spectra of TGM-D1 (A) or TGM-D2 (B) alone (red) overlaid with the spectrum of the same sample but with 1.2 equivalents of unlabeled TβRII added (blue). **C-E.** ^{1}H-^{15}N HSQC spectra of 0.03 mM ^{15}N TβRII alone (red) overlaid with the spectrum of the same sample, but with 1.2 equivalents of unlabeled TGM-D1 (C), TGM-D2 (D), or TGM-D3 (E) added (blue). Expansion of boxed region in panel E at intermediate titration points is shown below panel E. Spectra recorded in 25 mM sodium phosphate, 50 mM sodium chloride, 5% $^{2}H_2O$ pH 6.0, 310 K.

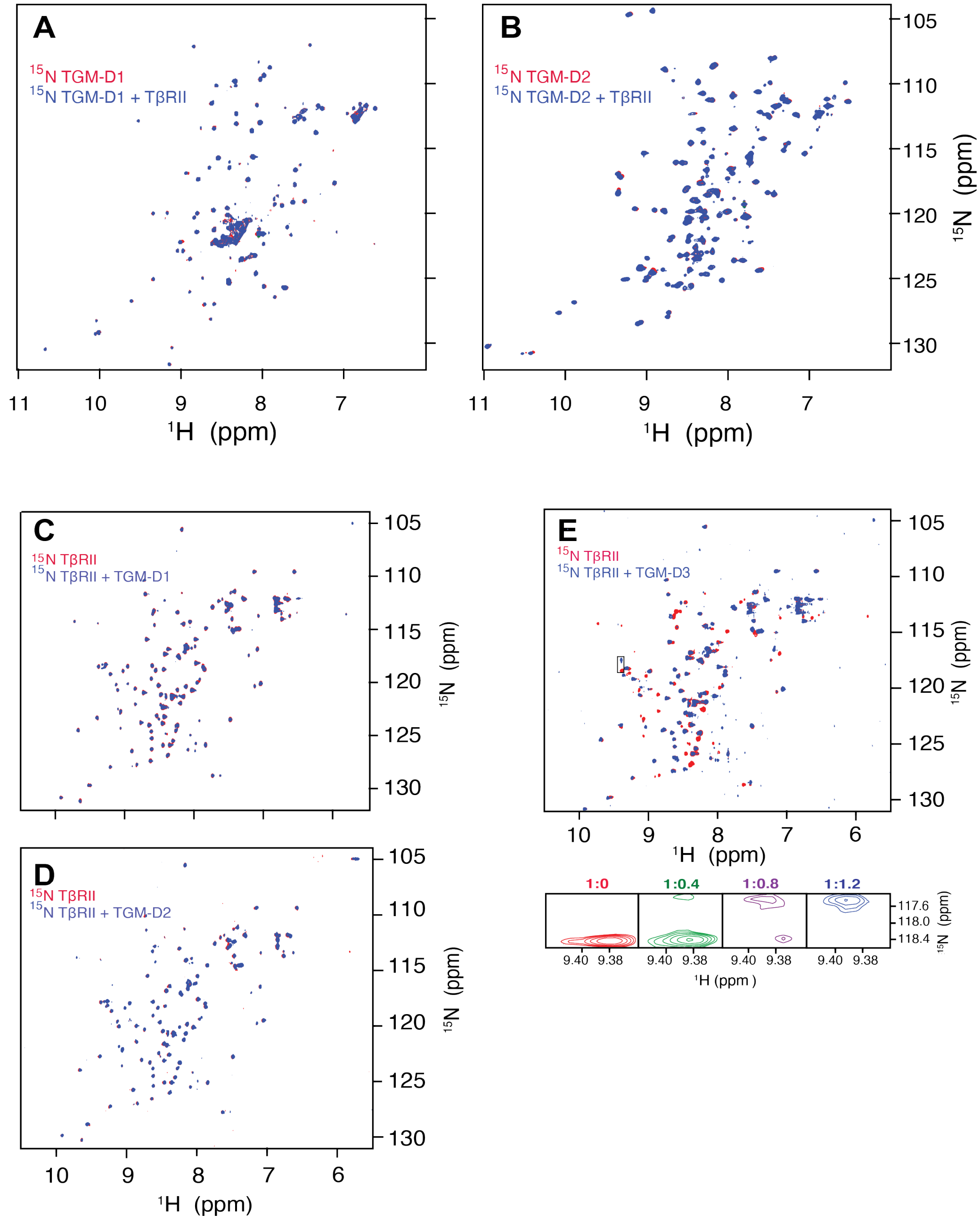

Figure S8. ^{1}H-^{15}N HSQC assignments of TβRII alone and as bound to TGM-D3. A. ^{1}H-^{15}N HSQC spectra of TβRII alone with peaks assigned. **B.** ^{1}H-^{15}N HSQC spectra of TβRII bound to TGM-D3 with peaks assigned. Dashed horizontal lines indicate sidechain -NH_2 resonances of Asn/Gln residues. Spectra recorded in 25 mM sodium phosphate, 50 mM sodium chloride, 5% $^{2}H_2O$ pH 6.0, 310K.

A

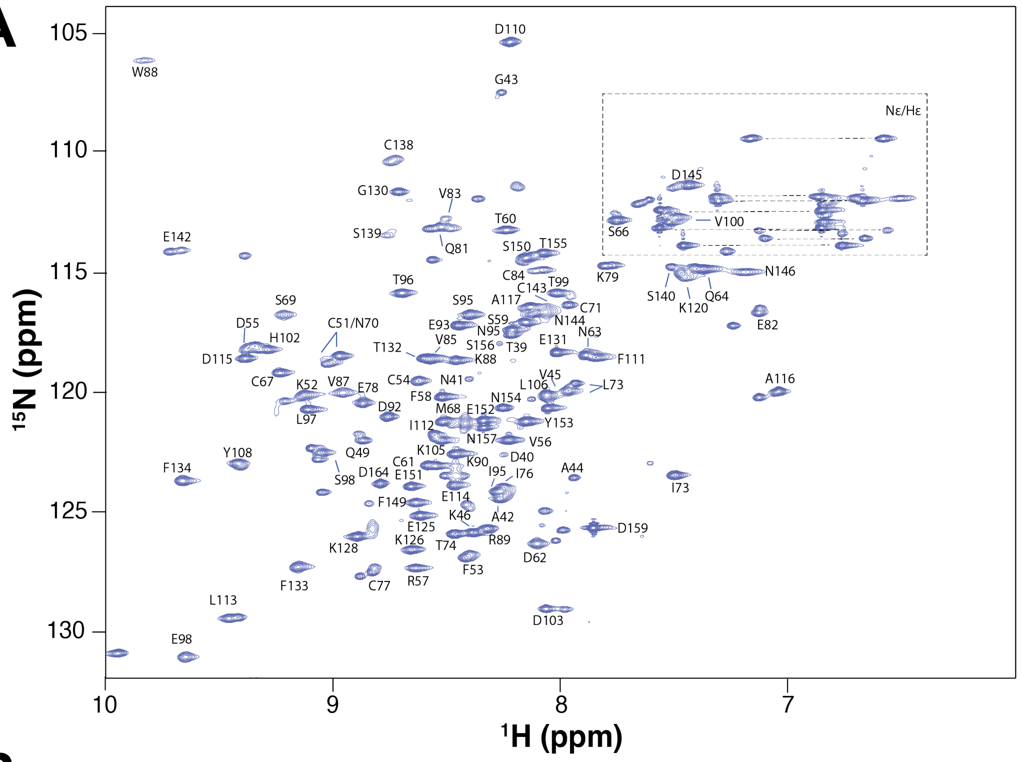

B

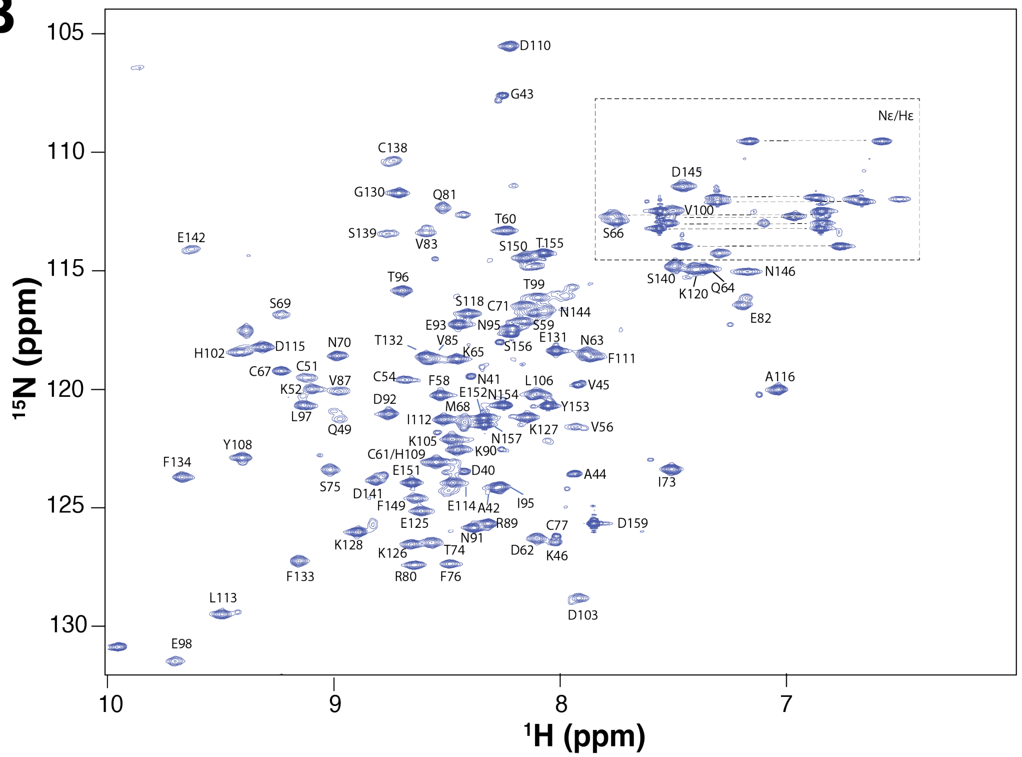

Figure S9. ^{1}H-^{15}N HSQC assignments of TGM-D3 alone and bound to TβRII. A-B. ^{1}H-^{15}N HSQC spectra of TGM-D3 alone (A) or bound to TβRII (B) with peaks assigned. Dashed horizontal lines indicate sidechain -NH_2 resonances of Asn/Gln residues.

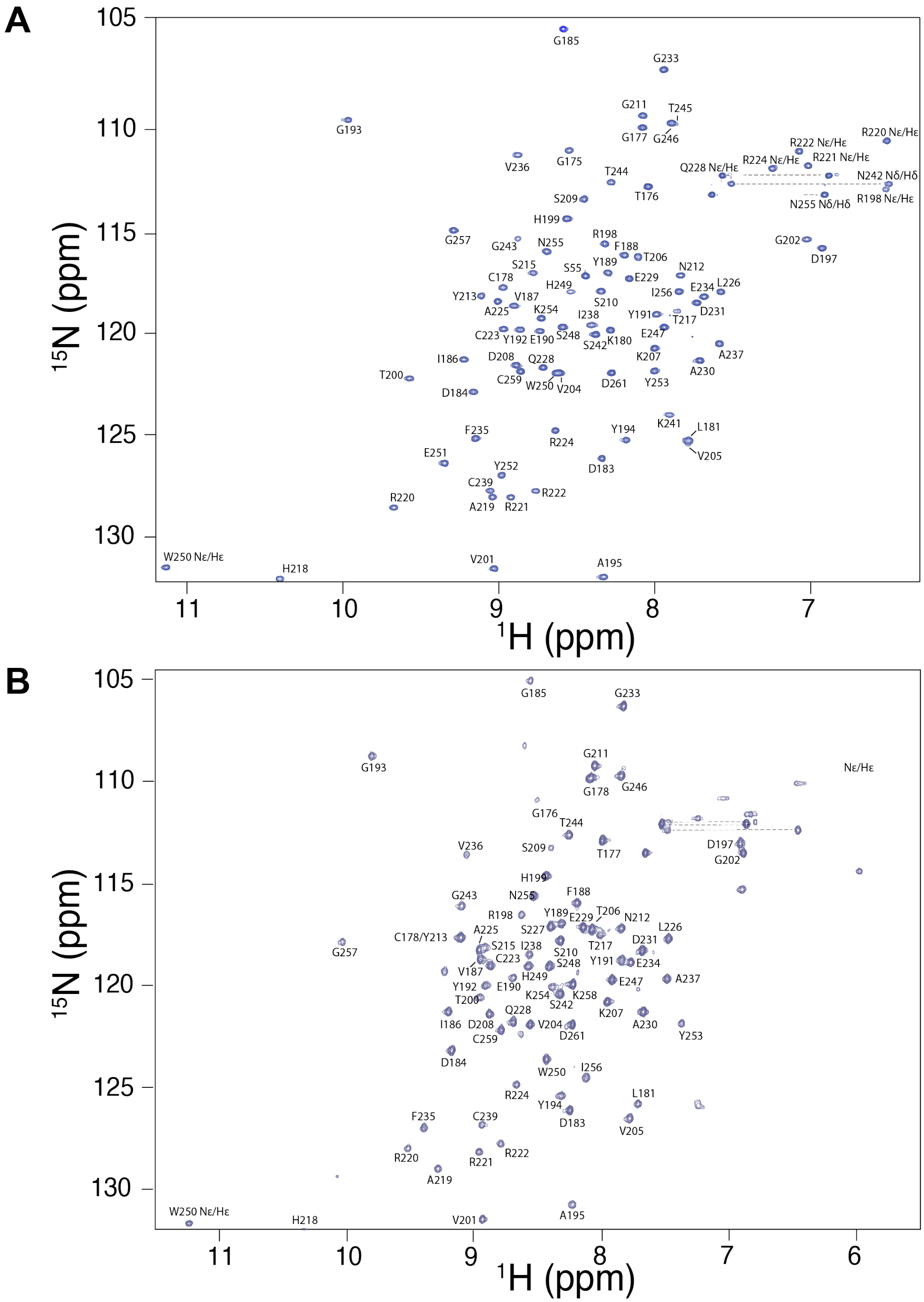

Figure S10: ^{1}H NMR spectra of TGM-D3 and TβRII single amino acid variants. A. ^{1}H NMR spectra of amide region (left) and methyl region (right) of TGM-D3 variants as compared to wild-type TGM-D3. **B.** ^{1}H NMR spectra of amide region (left) and methyl region (right) of TβRII variants as compared to wild-type TβRII. Spectra were collected in 25mM Na_2HPO_4, 150 mM NaCl, 0.02% NaN_3, pH 7.4 298K.

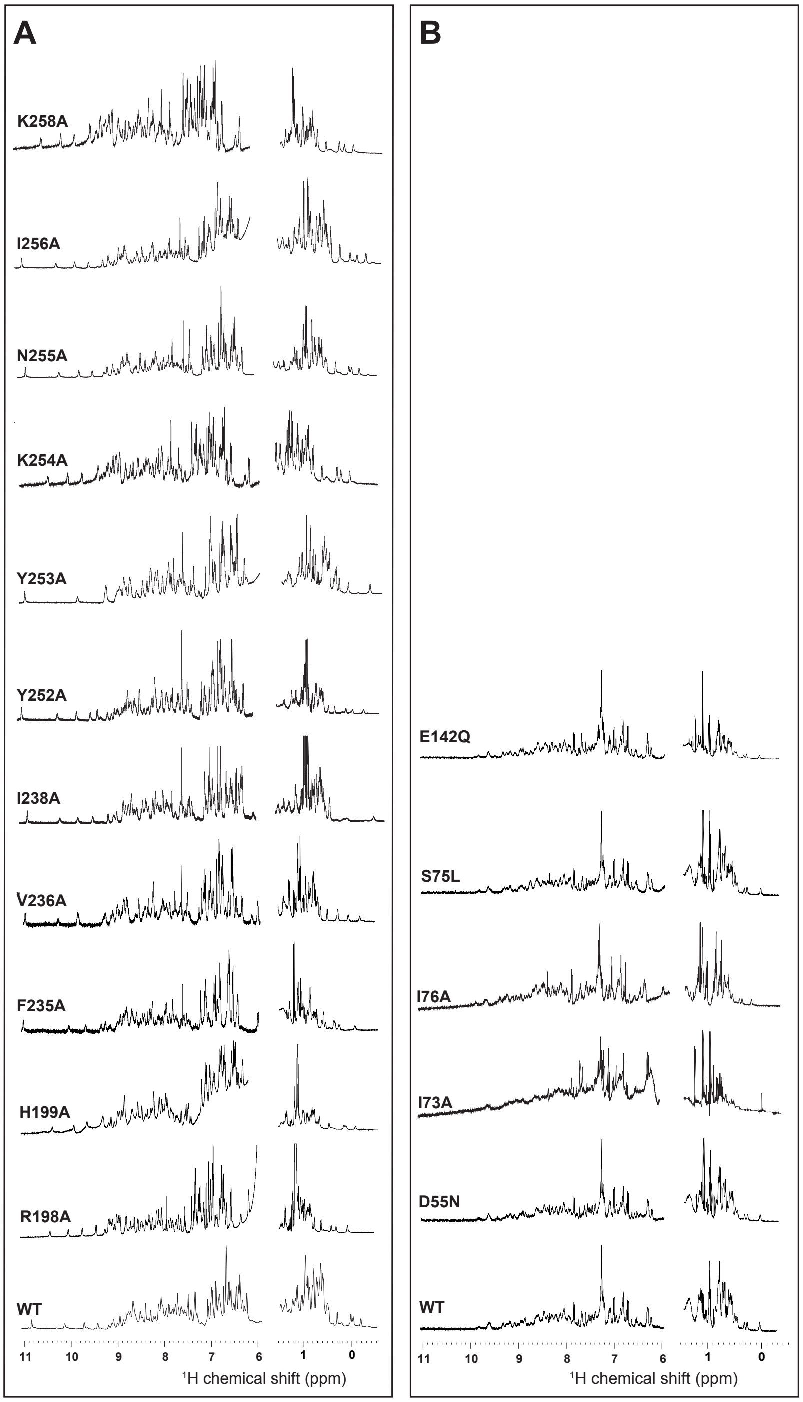

Figure S11. Binding of TβRII and TGM-D3 variants to their wild type counterparts. A-H. SPR sensorgrams obtained upon injection of TGM-D3 Arg[198]Ala (A), His[199]Ala (B), Phe[235]Ala (C), Val[236]Ala (D), Lys[254]Ala (E), Asn[255]Ala (F), Ile[256]Ala (G), and Lys[258]Ala(H), over immobilized TβRII. **I-N.** SPR sensorgrams obtained upon injection of TβRII WT (I), Asp[55]Asn (J), Ile[73]Ala (K), Ser[75]Leu (L), Ile[76]Ala (M), and Glu[142]Gln (N) over immobilized TGM-D3. Sensorgrams, obtained upon injection of a two-fold duplicate or triplicate dilution series of each construct are shown in black. Global fit of the sensorgrams to a 1:1 binding model are shown in orange. Black bars shown above the sensorgrams specify the injection period. Concentrations used and dissociation constants shown in the lower right.

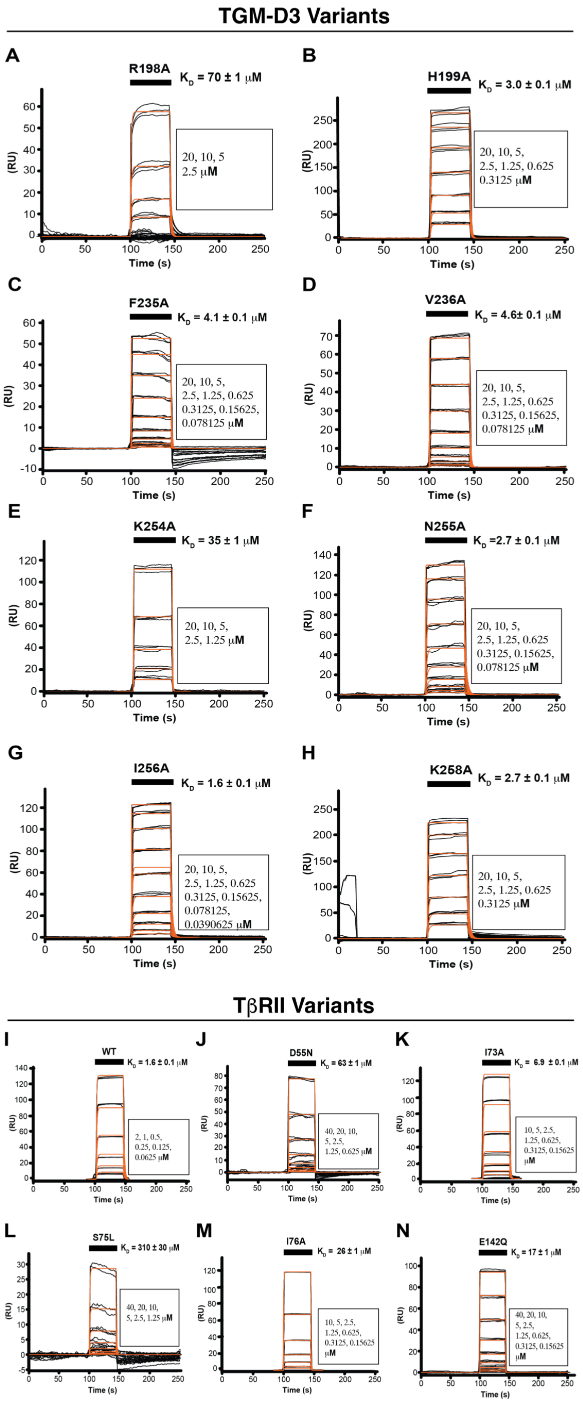

Figure S12. Alignment of TGM family domains. A-B. Alignment of TGM domain 3 with TGM domains 1, 2, 4, and 5 (A) and alignment of TGM domain 3 with the domain 3 of TGM-2, 3, 4, -5, -6, and -7 (B). Red indicates conserved residues while blue indicates similar residues. Overlaid on top are the secondary structural features of TGM-D3. Areas shaded in grey correspond to regions with composite shift perturbations of TGM-D3 due to TβRII binding greater than 0.1. Asterisks highlight residues of TGM-D3 which upon substitution led to a 4-fold or greater perturbation of the measured K_D value for binding TβRII.

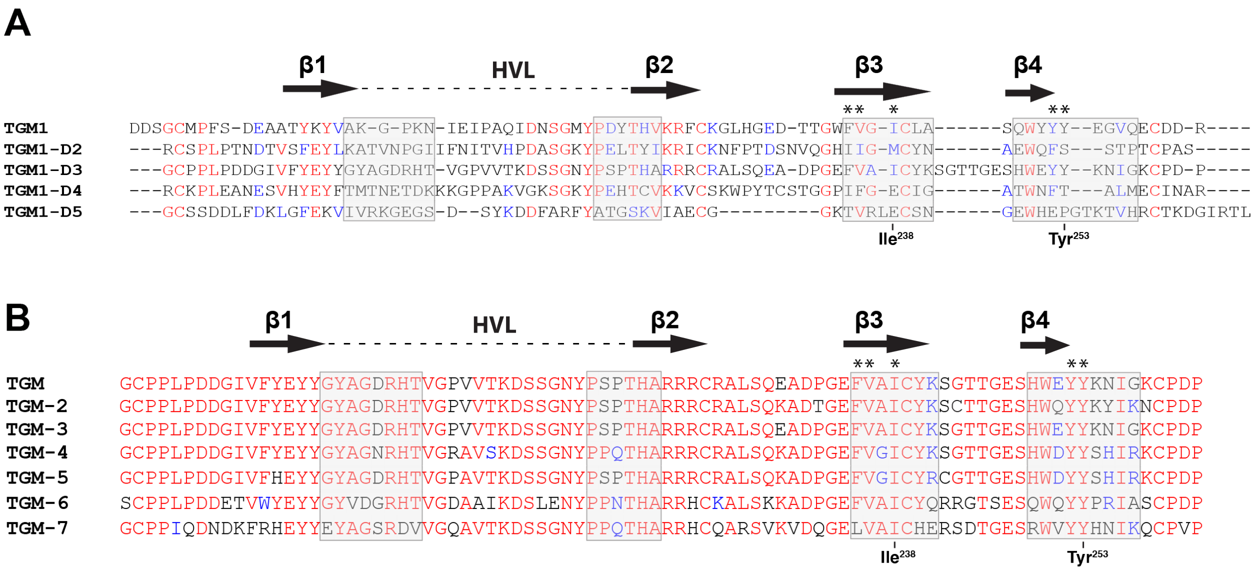

Supplement: Supplemental Tables S1–S5 and Figures S1–S12 [file mmc1.pdf]
